# PD-L1 CAR effector cells induce self-amplifying cytotoxic effects against target cells

**DOI:** 10.1136/jitc-2021-002500

**Published:** 2022-01-24

**Authors:** Malgorzata Bajor, Agnieszka Graczyk-Jarzynka, Katsiaryna Marhelava, Anna Burdzinska, Angelika Muchowicz, Agnieszka Goral, Andriy Zhylko, Karolina Soroczynska, Kuba Retecki, Marta Krawczyk, Marta Klopotowska, Zofia Pilch, Leszek Paczek, Karl-Johan Malmberg, Sébastien Wälchli, Magdalena Winiarska, Radoslaw Zagozdzon

**Affiliations:** 1 Department of Clinical Immunology, Medical University of Warsaw, Warszawa, Mazowieckie, Poland; 2 Laboratory of Immunology, Mossakowski Medical Research Institute, Polish Academy of Sciences, Warsaw, Poland; 3 Department of Immunology, Medical University of Warsaw, Warszawa, Poland; 4 Postgraduate School of Molecular Medicine, Medical University of Warsaw, Warsaw, Poland; 5 Laboratory for Cellular and Genetic Therapies, Medical University of Warsaw, Warsaw, Poland; 6 Department of Immunology, Transplantology and Internal Diseases, Medical University of Warsaw, Warszawa, Poland; 7 Doctoral School, Medical University of Warsaw, Warsaw, Poland; 8 Doctoral School of Translational Medicine, Centre of Postgraduate Medical Education, Warsaw, Poland; 9 Department of Bioinformatics, Institute of Biochemistry and Biophysics, Polish Academy of Sciences, Warsaw, Poland; 10 Department of Cancer Immunology, Radiumhospitalet, Oslo, Norway; 11 Department of Cellular Therapy, Oslo University Hospital, Oslo, Norway; 12 Department of Regenerative Medicine, The Maria Sklodowska-Curie National Research Institute of Oncology, Warsaw, Poland

**Keywords:** receptors, chimeric antigen, T lymphocytes, killer cells, natural

## Abstract

**Background:**

Immune checkpoint inhibitors and chimeric antigen receptor (CAR)-based therapies have transformed cancer treatment. Recently, combining these approaches into a strategy of PD-L1-targeted CAR has been proposed to target PD-L1^high^ tumors. Our study provides new information on the efficacy of such an approach against PD-L1^low^ targets.

**Methods:**

New atezolizumab-based PD-L1-targeted CAR was generated and introduced into T, NK, or NK-92 cells. Breast cancer MDA-MB-231 and MCF-7 cell lines or non-malignant cells (HEK293T, HMEC, MCF-10A, or BM-MSC) were used as targets to assess the reactivity or cytotoxic activity of the PD-L1–CAR-bearing immune effector cells. Stimulation with IFNγ or with supernatants from activated CAR T cells were used to induce upregulation of PD-L1 molecule expression on the target cells. HER2–CAR T cells were used for combination with PD-L1–CAR T cells against MCF-7 cells.

**Results:**

PD-L1–CAR effector cells responded vigorously with degranulation and cytokine production to PD-L1^high^ MDA-MB-231 cells, but not to PD-L1^low^ MCF-7 cells. However, in long-term killing assays, both MDA-MB-231 and MCF-7 cells were eliminated by the PD-L1–CAR cells, although with a delay in the case of PD-L1^low^ MCF-7 cells. Notably, the coculture of MCF-7 cells with activated PD-L1–CAR cells led to bystander induction of PD-L1 expression on MCF-7 cells and to the unique self-amplifying effect of the PD-L1–CAR cells. Accordingly, PD-L1–CAR T cells were active not only against MDA-MD-231 and MCF-7-PD-L1 but also against MCF-7-pLVX cells in tumor xenograft models. Importantly, we have also observed potent cytotoxic effects of PD-L1–CAR cells against non-malignant MCF-10A, HMEC, and BM-MSC cells, but not against HEK293T cells that initially did not express PD-L1 and were unresponsive to the stimulation. Finally, we have observed that HER-2–CAR T cells stimulate PD-L1 expression on MCF-7 cells and therefore accelerate the functionality of PD-L1–CAR T cells when used in combination.

**Conclusions:**

In summary, our studies show that CAR-effector cells trigger the expression of PD-L1 on target cells, which in case of PD-L1–CAR results in the unique self-amplification phenomenon. This self-amplifying effect could be responsible for the enhanced cytotoxicity of PD-L1–CAR T cells against both malignant and non-malignant cells and implies extensive caution in introducing PD-L1–CAR strategy into clinical studies.

## Introduction

Chimeric antigen receptor (CAR)-based strategies are one of the major breakthroughs in modern anticancer therapies. A crucial issue in designing CAR-based therapy is selecting a specific and safe target to ensure a proper balance between strength and precision. In the early studies, CD19, a surface marker of B cells, was identified as one of the most promising molecules for such interventions, as it is uniformly expressed on most B-cell-derived malignancies. To date, five CAR-based strategies have been approved by the U.S. Food and Drug Administration (FDA) for hemato–oncological applications. However, while CAR-based approaches have been applied in hematological malignancies with considerable success, their effectiveness in solid tumors treatment is modest at best. One of the reasons for this phenomenon is the immunosuppressive environment at the tumor site, which can be mediated by the expression of immune checkpoint molecules, such as programmed death-ligand 1 (PD-L1, CD274) acting on its cognate receptor PD-1 (CD279) on the immune effector cells.^1^ PD-L1 can be present either directly on cancer cells or on other cells in the tumor microenvironment (TME),^2 3^ making TME a formidable opponent of CAR T-based therapies. This has brought an idea of combining anti-PD-L1 approaches with CAR-based treatment.^3 4^ Thus, the anti-PD-1/PD-L1 targeting has indeed been attempted by numerous research groups in order to increase the potency of CAR T-based approaches (reviewed by Tang *et al* and Yoon *et al*).^4 5^ An extension of this idea was the generation of PD-L1-targeting CAR constructs. Accordingly, the PD-1- or atezolizumab-based CARs were very recently reported to act against cancer cells expressing high amounts of PD-L1. Moreover, in addition to direct killing of the PD-L1-expressing cancer cells, the PD-L1–CAR-bearing immune effector cells have shown the ability to reshape the TME,^6^ by eliminating the tumor-infiltrating macrophages and neutrophilic and monocytic myeloid cells endogenously expressing high levels of PD-L1.^7^ Additionally, multiple healthy cells were demonstrated to express detectable levels of PD-L1 in steady state or on induction.^8^

In the current work, we have observed that PD-L1–CAR cells stimulate PD-L1 expression on target tumor PD-L1^low^ cells. We have also investigated how non-malignant cells, with initially high or low/null expression of PD-L1, respond to the PD-L1–CAR-bearing effectors. Consequently, we present new data on the responsiveness in vitro and in vivo of both malignant and non-malignant PD-L1^low^ target cells to PD-L1–CAR effector cells due to the unique self-amplifying ability of this therapeutic approach. Our results help to understand the mechanisms of potential non-selectivity of the PD-L1–CAR regarding the on-target, off-tumor effect. Moreover, we demonstrate that the PD-L1 induction phenomenon after treatment with CAR T cells is also observed for human epidermal growth factor receptor 2(HER-2)–CAR. In this setting, PD-L1–CAR could be further explored to potentiate the antitumor activity of CAR-based approaches in the therapy of solid tumors. Hereby, we propose a sequential approach involving the prior application of HER-2–CAR as an exemplary tumor-specific CAR, promoting the functionality of PD-L1–CAR when used in combination.

## Materials and methods

All materials and methods are available in the online supplemental file.

10.1136/jitc-2021-002500.supp1 Supplementary data

## Results

### PD-L1 as a target for CAR-based therapy

**Figure 1:**
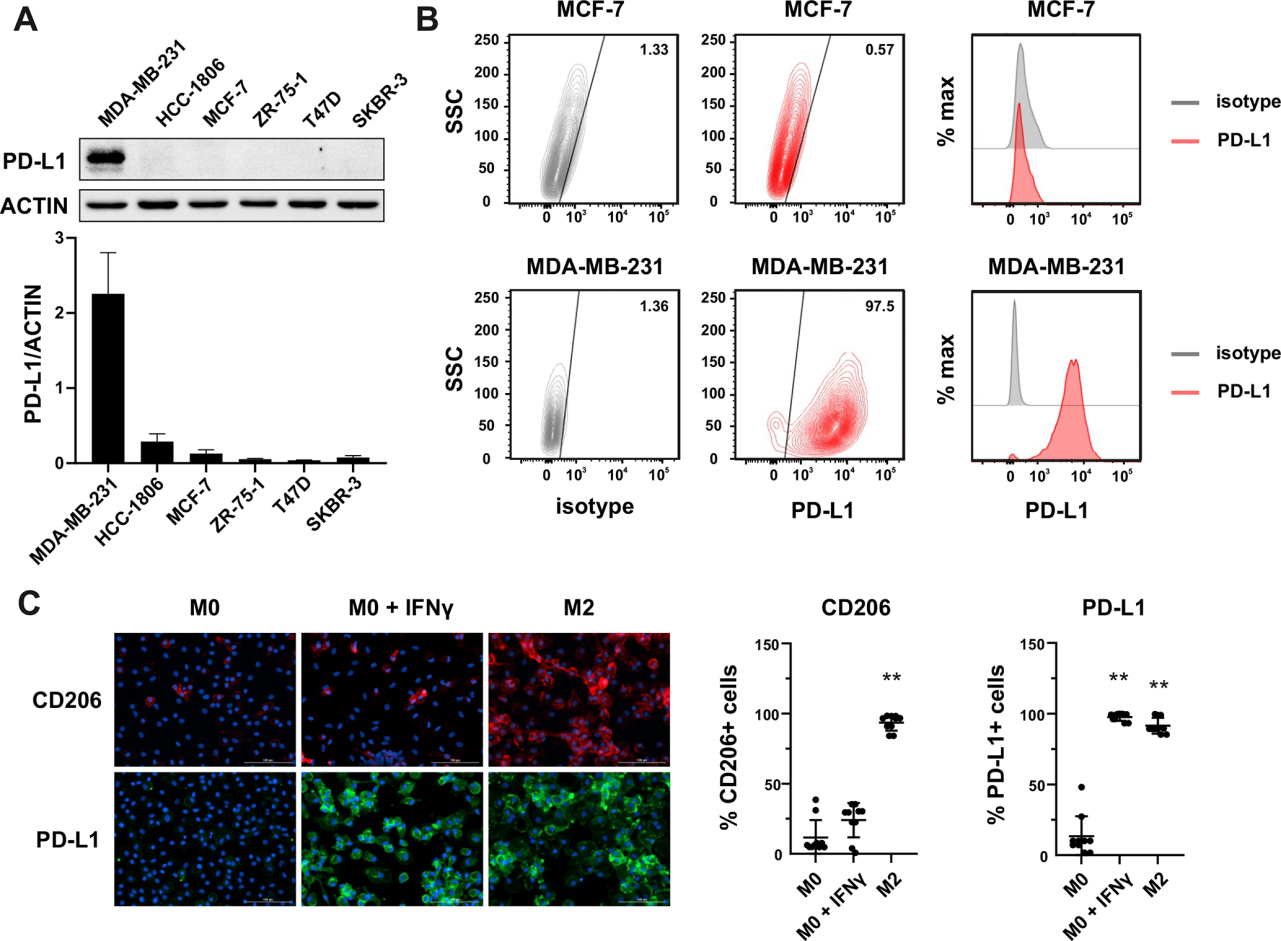
Expression of PD-L1 in breast cancer cell lines and macrophages. (A) The representative western blot analysis of PD-L1 expression in triple-negative (MDA-MB-231, HCC-1806), ER-positive (MCF-7, ZR-75-1, T47D), and HER-2-positive (SKBR-3) breast cancer cell lines (upper panel). β-actin was used as a loading control. The experiment was repeated three times. Bands were quantified by densitometry; the signal for PD-L1 band was normalized to the corresponding actin band (lower panel). (B) Representative density plots and histogram overlays illustrating PD-L1 expression (red) against a background from isotype control (gray) for MCF-7 (upper panel) and MDA-MB-231 (lower panel) breast cancer cell lines using flow cytometry. The staining was performed using an anti-PD-L1 antibody (cat. no. 12-5983-42, eBioscience, clone MIH1, dilution 1:100). Numbers on the density plots indicate the percentage of PD-L1-positive cells. The experiment was repeated at least three times. (C) PD-L1 expression in macrophage subpopulations (M0, M0+IFNγ, M2) detected by immunocytohistochemistry assay using Cytation 1 Cell Imaging Multi-Mode Reader (BioTek, Agilent). PD-L1 positively stained cells were detected using an anti-PD-L1 antibody (clone MIH1, cat. no. 14-5983-82, eBioscience, dilution 1:100) and are marked in green; red shows CD206-positive cells (cat. no. AF2534, R&D Systems, dilution 1:100). The signal was developed using AF488-conjugated or AF647-conjugated secondary antibody, respectively, and nuclei were counterstained with DAPI (blue). Bar graphs represent the quantitative analysis of either CD206 or PD-L1 expression in different macrophage subpopulations as a percentage of all cells. Data aggregated from three experiments performed in duplicates with two to four donors in each experiment (n=10). Bars represent the mean value±SD. The normality was checked using the Shapiro-Wilk test. The p values derived from Wilcoxon test (comparing to control): **p<0.01. PD-L1, programmed death-ligand 1, HER2, human epidermal growth factor receptor 2, IFNγ, interferon γ, DAPI, blue-fluorescent DNA stain (4′,6-diamidino-2-phenylindole).

Since PD-L1 within tumor mass can be observed on both tumor cells and TME cells, we performed screening experiments to determine the PD-L1 expression in various cell types. In the first step, we identified the PD-L1-positive tumor cells in a set of 18 cell lines derived from various cancers by western blotting. As shown in figure 1A and online supplemental figure 1A, detectable levels of PD-L1 protein expression were observed in a proportion of cell lines derived from breast cancer, particularly of the triple-negative phenotype (MDA-MB-231—PD-L1^high^, HCC-1806—PD-L1^moderate/low^), ovarian cancer (OvCa3, MDAH), Hodgkin’s lymphoma (SUO-HD1, HDLM-2), or malignant melanoma (M257). In a further study, we decided to focus on breast cancer models, as the expression of PD-L1 protein in this cancer significantly correlates with survival outcome in patients with mammary malignancies.^9 10^ Notably, our results of the variable expression of PD-L1 in breast cancer cell lines are in accordance with previous observations in primary mammary malignancies.^11^ Therefore, our data support the notion that a proportion of breast cancers, especially of triple-negative phenotype, could potentially be directly targeted by PD-L1–CAR. As the surface expression of the target molecule is crucial for the CAR efficacy, we have validated PD-L1 expression by flow cytometry using two anti-PD-L1 monoclonal antibodies: MIH-1 clone (figure 1B for MCF-7 and MDA-MB-231 and online supplemental figure 1B for HCC1806) and 29E.2A3 clone (online supplemental figure 1C). Consequently, MCF-7 cells (PD-L1^low/null^) and MDA-MB-231 cells (PD-L1^high^) were used as predominant models in our study.

10.1136/jitc-2021-002500.supp2 Supplementary data

Additionally, as a significant percentage of mammary tumors exhibit PD-L1 expression also in their stroma,^12^ in the next step, we evaluated the PD-L1 protein expression in macrophages, which are known as key contributors of tumor stroma formation.^13 14^ We have studied the PD-L1 expression using immunofluorescent microscopy in the M0, interferon γ (IFNγ)-stimulated M0, and M2 phenotypes; additionally, CD206 as a characteristic marker for the M2 phenotype was used. As shown in figure 1C, while in the M0 phenotype, the expression of PD-L1 protein was undetectable, the IFNγ-stimulated macrophages or those differentiated into M2 phenotype exhibited high intensity of PD-L1 staining. These findings indicate that the macrophages attributed to the intratumoral immunosuppressive microenvironment can constitute a potential target for the PD-L1–CAR strategy.

### Generation and expression of PD-L1–CAR in immune effector cells

**Figure 2:**
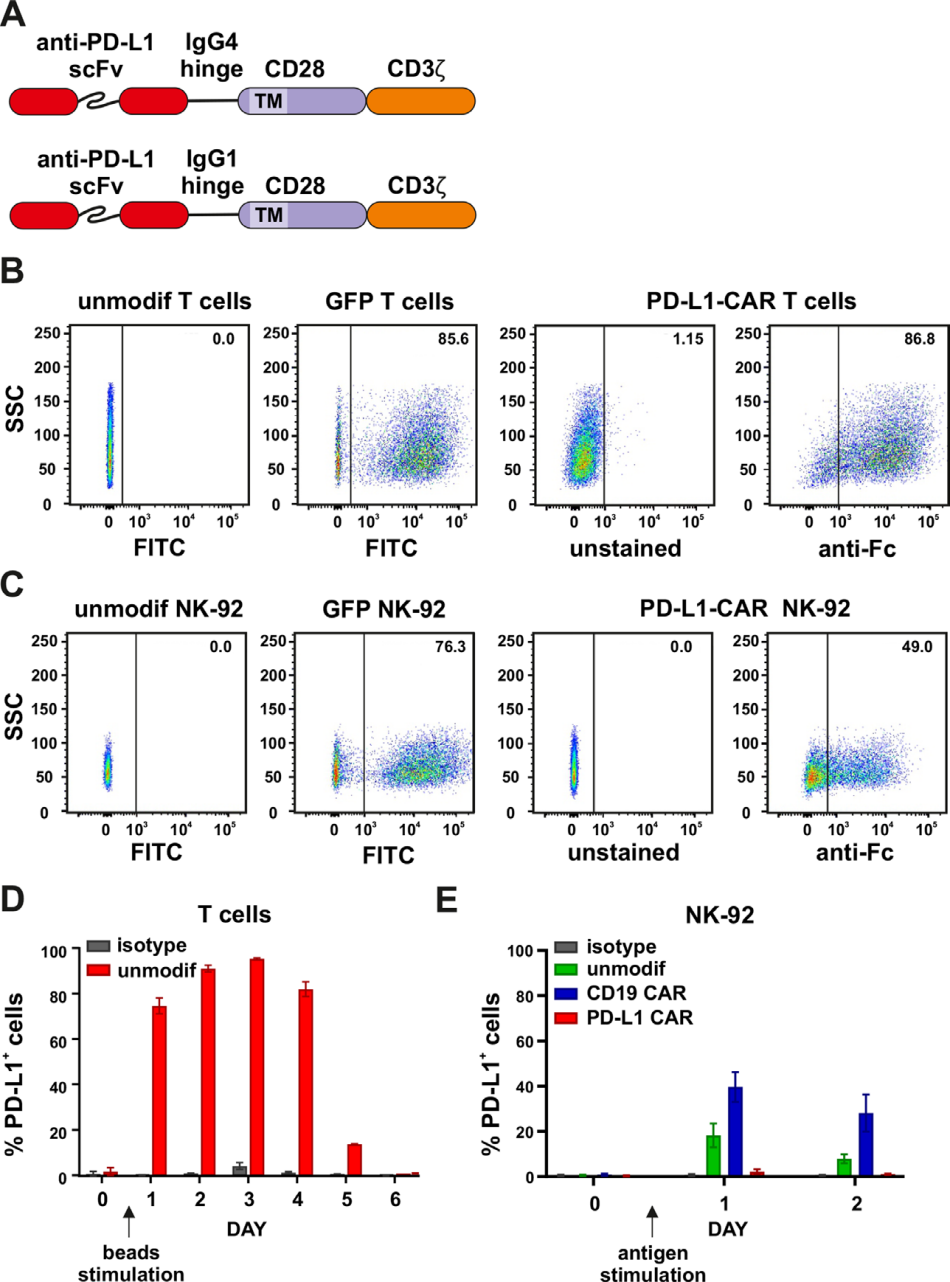
Generation and expression of PD-L1–CAR in immune effector cells. (A) The scheme depicting the modular structure of PD-L1–CARs used in the study (in detail described in the Materials and methods section). (B) Flow cytometry analysis of GFP (left panels) or PD-L1–CAR (right panels) expression in T cells after lentiviral transduction. GFP expression was detected in FITC channel and PD-L1–CAR expression was detected using anti-human IgG, Fcγ fragment specific antibody (cat. no. 109-606-098, Jackson ImmunoResearch). Numbers on the density plots indicate the percentage of PD-L1–CAR-positive cells. The experiments were repeated at least three times. (C) Flow cytometry analysis of GFP (left panels) or PD-L1–CAR (right panels) expression in NK-92 cells after lentiviral transduction was performed as described in (B). (D) PD-L1 expression on primary T cells. Bar graphs represent PD-L1 expression on unmodified effector cells T cells. T cells were cultivated in the presence of 100 U/mL of IL-2 alone (day 0) or together with human T-activator CD3/CD28 beads (days 1–6). Day 1 represents the first day after the stimulation of T cells with human T-activator CD3/CD28 beads. PD-L1 staining was performed on consecutive days using an anti-PD-L1 antibody (clone 29E.2A3, dilution 1:100). The experiment was repeated in duplicates two times. (E) PD-L1 expression on NK-92 cell line. Flow cytometry analysis of PD-L1 expression in NK-92, CD19–CAR NK-92, and PD-L1–CAR NK-92 cells following the stimulation with target Raji PD-L1 cells. The effector cells were coincubated with targets in a 1:1 E:T ratio. The PD-L1 expression on NK-92, CD19–CAR and PD-L1–CAR NK-92 cells was assessed 24 and 48 hours after the stimulation using an anti-PD-L1 antibody (clone MIH1, cat. no. 14-5983-82, eBioscience, diluted 1:100). Bar graphs represent the percentage of PD-L1-positive cells. The experiment was repeated three times. CAR, chimeric antigen receptor, PD-L1, programmed death-ligand 1, GFP, green fluorescent protein, FITC, fluorescein isothiocynate, scFV, single-chain variable fragment

We generated a new atezolizumab-based single-chain variable fragment (scFv) and combined it with a standard second-generation CAR backbone comprised of IgG4 hinge region, CD28 transmembrane and signaling portions, and the CD3ζ signaling domain (figure 2A). For the stable long-term exogenous expression, lentiviral transduction was used, and CAR expression was evaluated by the anti-Fc staining (figure 2B and online supplemental figure 2A). We also introduced PD-L1–CAR into NK cells by electroporation (online supplemental figure 2B) or into NK-92 cells by lentiviral transduction (figure 2C). NK-92 cells were further enriched in the PD-L1–CAR-positive population, up to approximately 90% (online supplemental figure 2C) by sorting. In subsequent experiments, we used both T cells or NK cells based models of CAR-engineered effector cells to determine the antitumor activity of the PD-L1 CAR-based approaches.

Importantly, we observed that the stimulation of T cells with CD3/CD28 beads prior to modification led to the PD-L1 upregulation already after 24 hours, which remained high until day 4 and decreased back to initial expression ranges by day 6 (figure 2D), posing the potential risk of the fratricidal killing. To address this issue, the CD19–CAR T (online supplemental figure 3A) or PD-L1–CAR T cells, 48 hours after lentiviral transduction with respective CAR-encoding vectors, were stimulated with the anti-CD3/anti-CD28-coated beads, and the dynamics of PD-L1 surface expression was assessed by flow cytometry. As shown in online supplemental figure 3B, the dynamics of PD-L1 expression in CD19–CAR T cells resembled the ones in primary T lymphocytes (figure 2D). However, no apparent induction of PD-L1 expression was detected in PD-L1–CAR T cells. In order to get a closer insight into this subject, we further documented the general capability of fratricidal killing by PD-L1–CAR T cells in coculture experiments with genetically unmodified activated T cells (online supplemental figure 3C). Our results clearly indicate that bead-stimulated unmodified T cells are indeed eliminated by PD-L1–CAR-bearing T cells and this effect is alleviated by atezolizumab. Furthermore, we performed western blotting to assess the general content of PD-L1 protein in CAR-bearing cells (online supplemental figure 3D) and the results were closely corresponding to the flow cytometry-based observations. Altogether, our data indicated that by expressing the PD-L1–CAR in T cells, we have here generated a unique population of T cells with suppressed expression of PD-L1 protein. Surprisingly, when we studied PD-L1 mRNA expression in these cells by qPCR (online supplemental figure 3E), the expression pattern was closely resembling the ones in unmodified and CD19–CAR T cells, that is, PD-L1–CAR T cells were clearly capable of inducing PD-L1 mRNA expression following stimulation. This suggests that the mechanism of PD-L1 protein suppression is post-transcriptional. We find this observation highly interesting from the biological point of view and worth exploring in future investigations. Moreover, we have carried out phenotyping of CD19- versus PD-L1-CAR-T cells on days 4 and 10 following transduction (online supplemental figure 3F). While we did detect differences in PD-L1 expression, in concordance with other results in our study, we did not see significant differences in other activation/exhaustion markers, apart from a change in PD-1 expression on day 4.

To compare, we also evaluated the NK-92 cell line (figure 2E) for the capability of inducing PD-L1 protein expression after target-specific stimulation. Again, as presented in figure 2E, we observed that unmodified (ie, naturally cytotoxic) or CD19–CAR-bearing NK-92 cells have responded to the target stimulus (ie, Raji–PD-L1 cells) with an increase of PD-L1 expression, while the PD-L1–CAR-bearing NK-92 cells retained the PD-L1 membrane presence at undetectable levels.

### The activity of PD-L1–CAR effector cells against tumor cells

**Figure 3:**
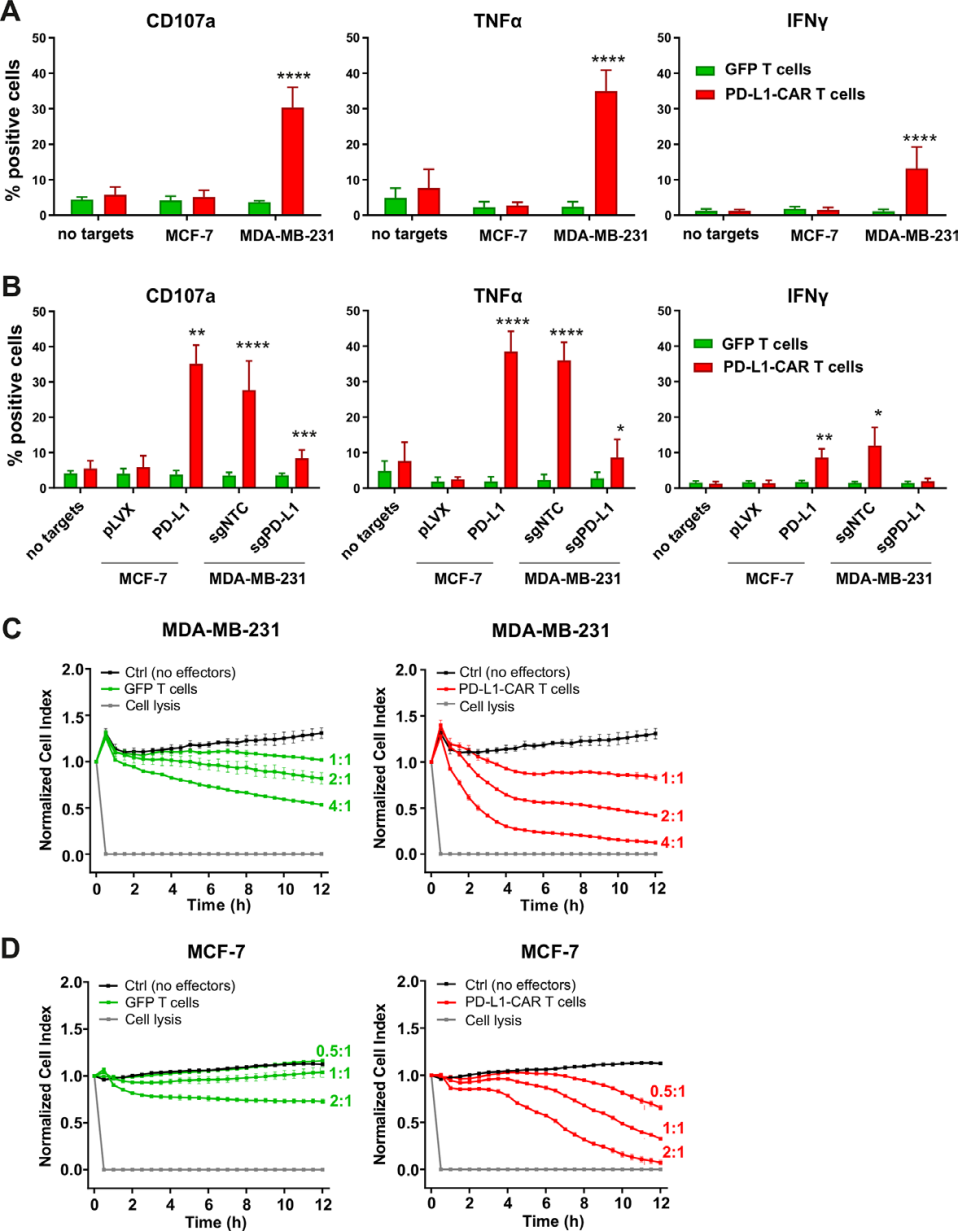
Cytokine production and degranulation by PD-L1–CAR T cells following stimulation with breast cancer cells. (A) Functional and cytokine release assays of PD-L1–CAR T cells targeted against MCF-7 (PD-L1^low/−^) or MDA-MB-231 (PD-L1^+^) cancer cell lines. Degranulation assay, assessed by CD107a staining (left panel), TNFα release (middle panel), and IFNγ release (right panel) were measured after 4 hours of coincubation of target and effector cells at the E:T ratio of 2:1. The experiment was repeated in duplicates three times. Bars represent the mean value±SD. The normality was checked using the Shapiro-Wilk test. The p values derived from unpaired t-test: ****p<0.0001. (B) Functional and cytokine release assays of PD-L1–CAR T cells targeted against MCF-7 pLVX (PD-L1^low/−^) and MCF-7 PD-L1 and MDA-MB-231 sgNTC (PD-L1^+^) or MDA-MB-231 sgPD-L1 (PD-L1^-^) cancer cell lines were assessed by flow cytometry. Degranulation assay, assessed by CD107a staining (left panel), and IFNγ release (right panel) were measured after 4 hours of coincubation of target and effector cells at the E:T ratio of 2:1. The experiment was repeated in duplicate three times (for IFNγ release by MCF-7 for two times). Bars represent the mean value±SD The p values derived from unpaired t-test or Mann-Whitney test depending on data distribution (*p<0.05, **p<0.01, ***p<0.001, ****p<0.0001). (C) The potential of killing tumor cells by control (pSEW-GFP) and PD-L1–CAR T cells was measured by impedance analysis for MDA-MB-231 cells. Cancer cell lines were left to adhere and form a monolayer on the E plates for 24 hours. The next day, PD-L1–CAR T cells or control (pSEW-GFP) T cells were added to the monolayers for 12 hours at the indicated E:T ratios. Representative mean impedance curves from two wells were shown. The experiment was repeated in duplicates two times. (D) The potential of killing tumor cells by control (pSEW-GFP) and PD-L1–CAR T cells was measured by impedance analysis for MCF7 cells. Cancer cell lines were left to adhere and form a monolayer on the E plates for 24 hours. The next day, PD-L1–CAR T cells or control (pSEW-GFP) T cells were added to the monolayers for 12 hours at the indicated E:T ratios. Representative mean impedance curves from two wells were shown. The experiment was repeated in duplicates two times. CAR, chimeric antigen receptor, PD-L1 - programmed death-ligand 1, TNFα - tumor necrosis factor α, IFNγ - interferon γ, GFP - green fluorescent protein, SSC - side scatter

To investigate the efficacy of a newly generated PD-L1–CAR against breast cancer cell lines, PD-L1–CAR T cells or NK cells were incubated with target MDA-MB-231 (PD-L1^high^) or MCF-7 (PD-L1^low/null^) cells. Degranulation of CAR T cells assessed by CD107a staining and tumor necrosis factor α (TNFα) or IFNγ production was compared with the effector cells transduced with green fluorescent protein(GFP)-encoding control vector. As expected, only incubation with MDA-MB-231 induced both potent degranulation and cytokines’ production by PD-L1–CAR T cells (figure 3A), PD-L1–CAR–NK-92 cells (online supplemental figure 4A), or PD-L1–CAR–NK cells within 4 hours (online supplemental figure 4B). To verify the specificity of the new PD-L1–CAR, we generated the PD-L1-knockout (KO) derivatives of MDA-MB-231 cell line (MDA-MB-231-sgPD-L1; online supplemental figure 4C, D) and also the PD-L1-overexpressing derivative of MCF-7 cell line (MCF-7-PD-L1; online supplemental figure 4E, F). When incubated with PD-L1–CAR T cells, MDA-MB-231-sgPD-L1 cells significantly decreased the effector cells' degranulation and cytokines’ production, and the opposite effect was observed with MCF-7-PD-L1 cells (figure 3B for PD-L1–CAR T cells and online supplemental figure 4G for PD-L1–CAR–NK-92 cells).

**Figure 4:**
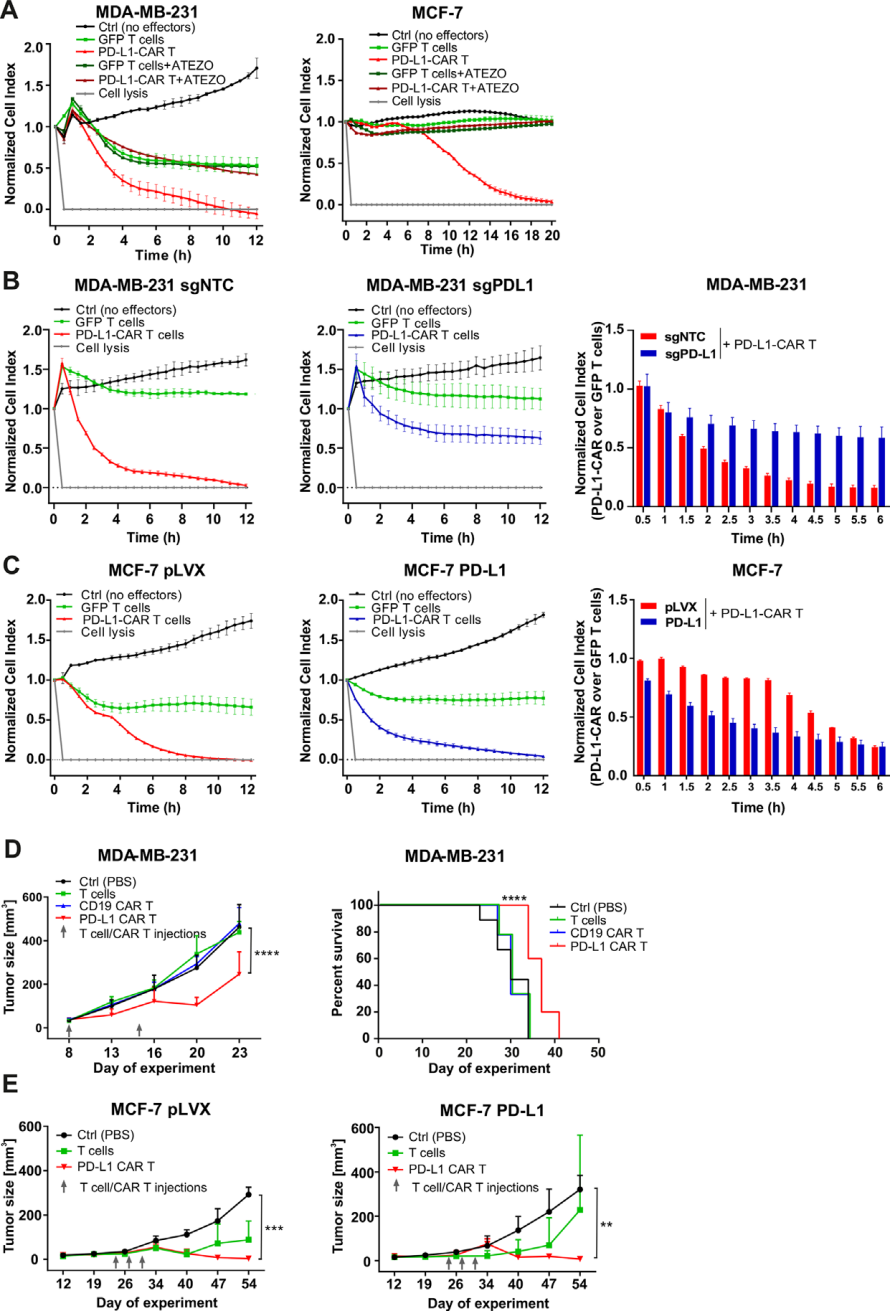
The efficacy of PD-L1–CAR T cells against breast cancer cells with different PD-L1 protein expression. (A) The killing potential of PD-L1–CAR T cells against MDA-MB-231 (left panel) or MCF-7 (right panel) breast cancer cells was measured by impedance analysis. Cancer cell lines were seeded on the E plates and left to adhere and form a monolayer for 24 hours. The next day, PD-L1–CAR T cells or control (pSEW-GFP) T cells were added to the monolayers at the E:T ratio of 2:1 (MDA-MB-231) and 1:1 (MCF-7) in the absence or presence of 0.4 mg/mL atezolizumab. The cultures were monitored for the next 12 hours. Representative mean impedance curves from two wells are shown. The experiment was repeated in duplicates three times. (B) The cytotoxic activity of PD-L1–CAR T cells against MDA-MB-231 sgNTC (left panel) and MDA-MB-231 sgPD-L1 (middle panel) cancer cell lines, at the E:T ratio of 2:1, were measured by impedance analysis. Samples were internally normalized for the cell index value measured before CAR T cells addition (Normalized Cell Index plots). Bar graph represents Normalized Cell Index values of quantification of PD-L1–CAR T-cell killing over GFP T cells control from 0.5 to 6 hours (right panel). Representative average impedance curves from two wells are shown. The experiment was repeated in duplicates two times. (C) Cytotoxic activity of PD-L1–CAR T cells against MCF-7 pLVX (left panel) and MCF-7 PD-L1 (middle panel) cancer cell lines at the E:T ratio of 1:1 were measured by impedance analysis. Samples were internally normalized for the cell index value measured before PD-L1–CAR T cells addition (Normalized Cell Index plots). Bar graph represents Normalized Cell Index values of quantification of PD-L1–CAR T-cell killing over GFP T cells control from 0.5 to 6 hours (right panel). Representative average impedance curves from two wells are shown. The experiment was repeated in duplicates two times. (D) Mean volume of MDA-MB-231 tumors after two rounds (days 8 and 15) of intratumoral administration of PBS (control), unmodified T cells, CD19–CAR or PD-L1–CAR T cells, +SD, two-way ANOVA test, ****p<0.0001, left panel. Corresponding Kaplan-Meier survival plot, analyzed by log-rank survival test, ****p<0.0001, right panel. The graphs present results summarized from two independent experiments, n=9–10. (E) Mean tumor size of MCF-7 pLVX (left panel, n=6–7) and MCF-7 PD-L1 (right panel, n=4–6). Mice were treated with PBS (control), unmodified T cells, or PD-L1–CAR T cells on days 24, 27, and 30, +SD, two-way ANOVA test, **p<0.01, ***p<0.001. CAR, chimeric antigen receptor, PD-L1, programmed death-ligand 1, PBS, phosphate-buffered saline, ANOVA - analysis of variance, GFP - green fluorescent protein.

To directly assess the cytotoxicity of PD-L1–CAR T cells, we performed real-time cell assays (RTCA) with MDA-MB-231 or MCF-7 cells. Surprisingly, a potent, E:T ratio-dependent, cytotoxic effect was seen against both types of cells, although it was delayed in the case of MCF-7 cells (figure 3C vs D, right panels, for MDA-MB-231 and MCF-7 cells, respectively). To confirm, that the delayed effectiveness of PD-L1–CAR T cells against PD-L1^low^ target cells is not unique for MCF-7, we have used another PD-L1^low^ cell line, namely T47D cells. As presented in online supplemental figure 4H, T47D cells were also successfully eliminated by PD-L1–CAR T cells, again with the cytotoxicity onset delayed by approximately 4–6 hours. Because of these surprizing observations, we further verified the specificity of PD-L1–CAR and used atezolizumab in order to block the binding of PD-L1–CAR T to its target competitively. Incubation with atezolizumab abrogated the cytotoxic effects of PD-L1–CAR T cells against the MDA-MB-231 (figure 4A, left panel) and also against MCF-7 cells (figure 4A, right panel). Moreover, in agreement with our previous results, the cytotoxicity of PD-L1–CAR T cells against the MDA-MB-231-sgPD-L1 cells was strongly diminished in the RTCA assay as compared with the MDA-MB-231-sgNTC controls (figure 4B), further confirming the specificity of PD-L1–CAR.

The introduction of PD-L1 into the MCF-7 line led to a rapid eradication by PD-L1 CAR cells (figure 4C, middle panel). Corroborating the initial observation in the parental MCF-7 (figure 3D), also the MCF-7-pLVX controls were eradicated although again with a slight delay (figure 4C, left). Indeed, the latency in the appearance of the CAR-mediated killing in MCF-7- PD-L1^low/null^ types of cells suggests that PD-L1 molecule may become upregulated on the MCF-7-pLVX cells with time during the coincubation with CAR-bearing cells.

To establish whether the observations of PD-L1–CAR effectiveness are reflected in the in vivo settings, we have carried out investigations in the MDA-MB-231-derived or MCF-7-derived human-to-mouse tumor xenograft models (figure 4D, E, respectively). First, we have observed that PD-L1–CAR T cells, but not the control treatment (ie, phosphate-buffered saline (PBS), unmodified T cells, and CD19–CAR T cells), induced significant retardation of MDA-MB-231 tumor growth (figure 4D, left-hand panel and online supplemental figure 4I) and prolonged mouse survival (as assessed by reaching the predefined tumor volume; figure 4D, right-hand panel). Importantly, we have also observed induction by PD-L1–CAR T cells of retardation in tumor growth in the models of both MCF-7-pLVX (figure 4E, left-hand panel and online supplemental figure 4J, left-hand panel) and MCF-7-PD-L1 xenografts (figure 4E, right-hand panel and online supplemental figure 4J, right-hand panel), which confirms that PD-L1–CAR can be effective against initially PD-L1 ^low/null^ cells in a growing in a living organism.

Altogether, the results described in the current subsection prompted us to elucidate further the mechanisms and dynamics of PD-L1 upregulation on target cells following their interaction with CAR-modified effector cells.

### Induction of PD-L1 expression on the target cells

**Figure 5:**
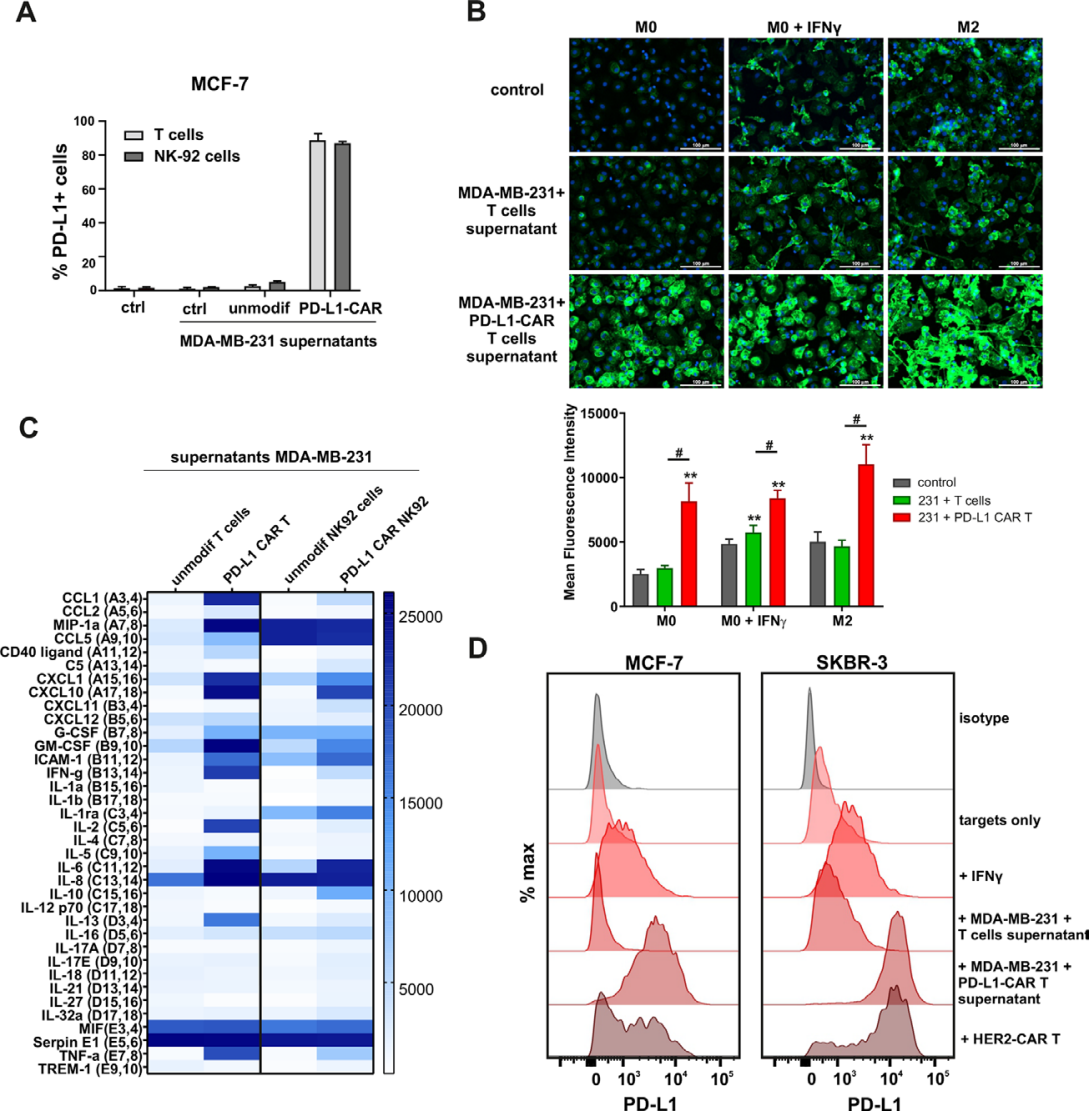
Induction of PD-L1 expression on the target cells. (A) PD-L1 expression induced by supernatant from activated CAR T or CAR–NK-92 cells on cancer cells was assessed by flow cytometry. The control (only medium) and conditioned supernatants from the 24 hours coincubation cultures of control (unmodified) or PD-L1–CAR T/NK-92 cells with the target MDA-MB-231 cells were transferred onto the culture of MCF-7 and incubated for 48 hours. Next, PD-L1 surface presence was assessed using anti-PD-L1 antibody (clone MIH1) by flow cytometry. The experiment was repeated three times. (B) Representative images of different subpopulations of macrophages (M0, M0+IFNγ, M2) stained for PD-L1 assessed by immunocytochemistry assay using Cytation 1 Cell Imaging Multi-Mode Reader (BioTek, Agilent). Macrophages were incubated with 10 ng/mL IFNγ or IL-4 and IL-10 (for M2) for 5 days before staining (every 2-day fresh portion of cytokines was added). The control (only medium) and conditioned supernatants from the 24 hours coincubation cultures of control (unmodified) or PD-L1–CAR T cells with the target MDA-MB-231 cells were transferred onto different subpopulations of macrophages and incubated for 48 hours. Next, PD-L1 surface presence was assessed using anti-PD-L1 antibody (clone MIH1, cat. no. 14-5983-82, eBioscience, diluted 1:100). The signal was developed using AF488-conjugated secondary antibody (green), and nuclei were counterstained with DAPI (blue), scale bar: 100 µm. The background fluorescence was removed, and the low threshold for green fluorescence was set to create a mask of the area covered by macrophages. Bar graphs represent the mean fluorescent intensity of PD-L1 within the thresholded area. Data aggregated from three experiments performed in duplicates with two to four donors in each experiment (n=8–10). Bars represent the mean value±SD. Normality was checked using the Shapiro-Wilk test. The p values derived from Student’s t-test or Wilcoxon test (comparing to control), depending on data distribution: **p<0.01. Two-sample Kolmogorov-Smirnov test was used to calculate statistics between T cells and CAR T groups: #p<0.01. (C) The relative levels of human cytokines in supernatants collected from control (unmodified T cells and NK-92 cells) or PD-L1–CAR T cells and NK-92 cells cocultured with MDA-MB-231 breast cancer cells for 24 hours at 1:1 E:T ratio were determined by cytokine array assay. The changes in the expression profile of 36 cytokines are presented on the heat map. The experiment was performed with two donors in duplicates. (D) Representative PD-L1 expression in MCF-7 (left-hand panel) or SKBR-3 (right-hand panel) cells after a 24 hours incubation in the presence of 20 ng/mL IFNγ, supernatants from coculture of unmodified T cells or PD-L1–CAR T cells with MDA-MB-231 and directly with HER2–CAR T cells at 1:1 E:T ratio. PD-L1 surface presence was assessed by flow cytometry using anti-PD-L1 antibody (clone MIH1). The experiment was performed three times in duplicates. CAR, chimeric antigen receptor, PD-L1, programmed death-ligand 1, IFNγ, interferon γ, NK- natural killer, HER2, human epidermal growth factor receptor 2, IL-4, interleukin 4

First, we addressed the question whether PD-L1 expression could be increased by antigen-activated CAR T cells in a self-amplifying mechanism. To answer this, we transferred the conditioned supernatants from the coincubation cultures of PD-L1–CAR T or PD-L1-NK-92 cells with the target MDA-MB-231 cells onto the MCF-7 or MDA-MB-231 cells and assessed PD-L1 surface expression by flow cytometry. As depicted in figure 5A, in MCF-7 cells conditioned supernatants from target-activated CAR T/NK-92 cells increased PD-L1 surface expression. Conversely, MDA-MB-231 cells presented uniformly high expression of PD-L1 regardless of the culture conditions, while PD-L1 KO (sgPD-L1) MDA-MB-231 cells were not able to express PD-L1, once incubated in the presence of conditioned supernatants from activated CAR T cells (online supplemental figure 5A). As activated T cells are known to secrete increased quantities of IFNγ, we hypothesized that the effect of CAR-conditioned medium depends on IFNγ production. We thus investigated the effects of IFNγ on surface PD-L1 levels in MDA-MB-231 and MCF-7 cells. IFNγ did not significantly change PD-L1 levels in MDA-MB-231 cells (online supplemental figure 5B, left panel). Simultaneously, MCF-7 cells’ surface PD-1 levels increased on IFNγ treatment (online supplemental figure 5B, right panel), however, to a much lesser extent than that observed on incubation with CAR T conditioned medium. Likewise, all three macrophage phenotypes studied (M0, IFNγ-stimulated M0, and M2) responded to the addition of the CAR T/targets conditioned medium with a very potent increase in PD-L1 expression, greatly exceeding those observed for IFNγ only (figure 5B and online supplemental figure 5C). In order to have an insight into the composition of CAR T/NK-92 conditioned media, we performed cytokine arrays, in which we identified a set of cytokines strongly upregulated after antigen-mediated activation of CAR T/NK-92 cells when compared with unmodified T or NK-92 cells, respectively (figure 5C and online supplemental figure 5D). From these experiments, we concluded that CAR T/NK-92 cells, on target-specific activation, produce considerable amounts of cytokines and chemokines that collectively are potent stimulators of PD-L1 expression and can be responsible for the observed self-amplifying cytotoxic activity of PD-L1–CAR-bearing cells. Since PD-L1–CAR T cells rapidly eliminate the target cells with induced PD-L1 expression cells (online supplemental figure 6A-C), we examined the full extent of bystander PD-L1 induction using HER-2–CAR T cells incubated with HER-2-positive target tumor. As shown in figure 5D (left panel), we observed a potent induction of PD-L1 expression on MCF-7 cells on incubation with HER-2–CAR T cells and an even stronger induction in the SKBR-3 cell line (figure 5D, right panel), most probably due to the higher abundance of HER-2 in the latter (online supplemental figure 6D).

In summary, we conclude that the induction of PD-L1 occurring during the incubation of target cells with CAR T cells is a universal phenomenon. Moreover, PD-L1–CAR T cells can act on their targets in a ‘rolling snowball’ mode. The cytotoxic effects against PD-L1^low^ targets can be self-amplifying and spreading to the surroundings due to CAR T-induced, in a juxtacrine and/or paracrine manner, increase of PD-L1 molecule expression on the surface of the target and bystander cells, respectively. To the best of our knowledge, such a self-amplification phenomenon is unique among the CAR-based strategies. From our understanding, it can act as a double-edged sword. A positive consequence for the therapy of tumors would be a markedly expanded potential spectrum of malignancies targeted with the PD-L1–CAR-based therapies. However, since the PD-L1^low^ cells can become targets for PD-L1–CAR cells, it becomes important to investigate whether such therapies are toxic against non-malignant cells.

### Expression of PD-L1 in non-malignant cells

**Figure 6:**
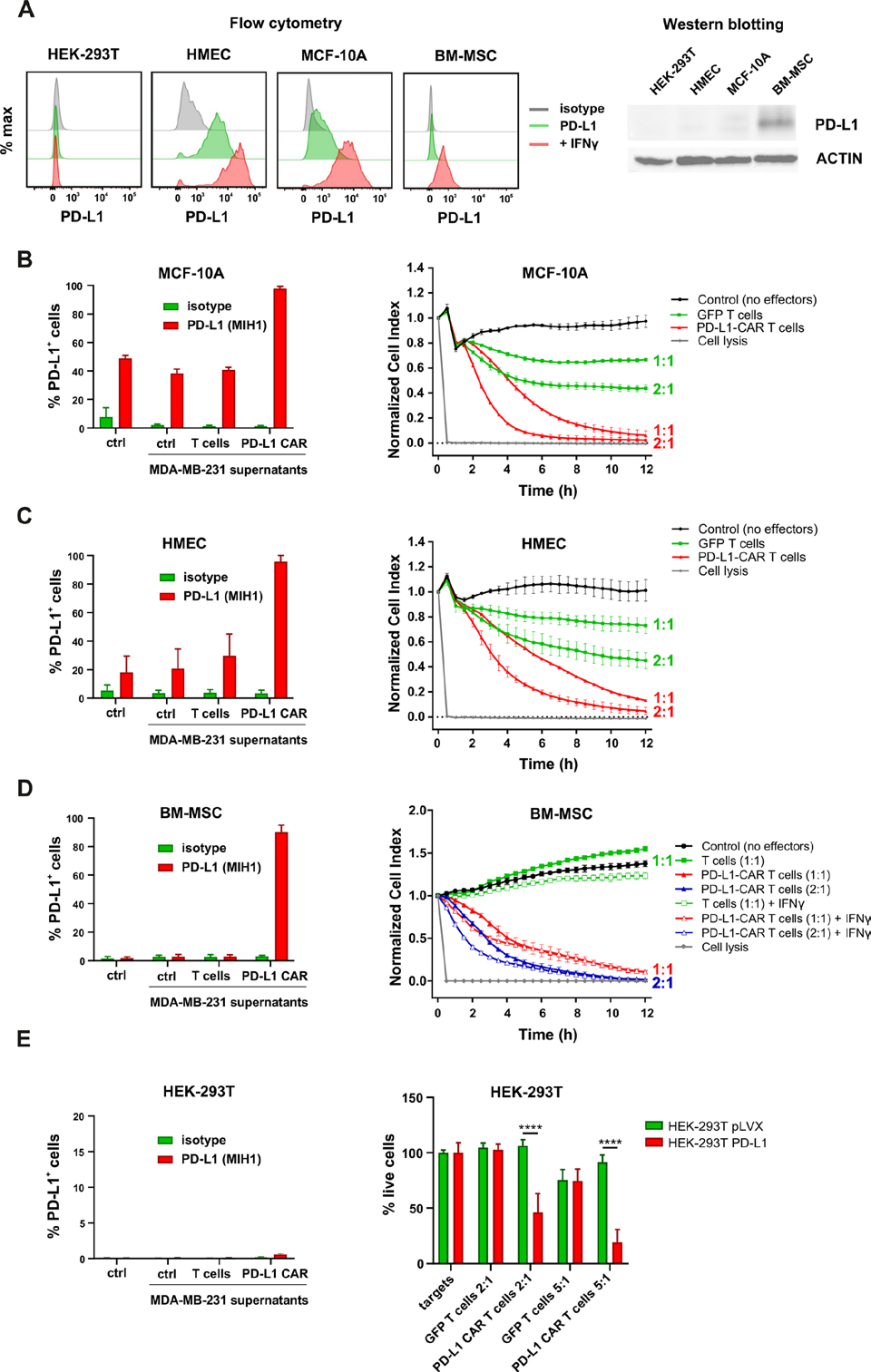
Expression of PD-L1 and PD-L1–CAR T mediated cytotoxicity in non-malignant cells. (A) IFNγ induced expression of PD-L1 on HEK293T cell line derived from human embryonic kidney cells and non-malignant cells (HMEC, MCF-10A, and bone marrow-derived mesenchymal stem cells (BM-MSC)) assessed by flow cytometry as presented on the left panel. PD-L1 staining was performed using anti-PD-L1 antibody (clone MIH1). The representative western blot analysis of PD-L1 expression in human embryonic kidney HEK293T cells and non-malignant mammary epithelial HMEC and MCF10A cells, and BM-MSC (right panel). β-actin was used as a loading control. The experiment was repeated three times. (B) PD-L1 expression induced on MCF-10A cells by activated CAR T cells (left panel) and RTCA-monitored cytotoxic activity of PD-L1 CAR T cells toward MCF-10A cells (right panel). The control (only medium) and conditioned supernatants from the 24 hours coincubation cultures in the presence of control (unmodified) T cells or PD-L1–CAR T cells with the target MDA-MB-231 cells were transferred onto the cultures of MCF-10A cell line and incubated for 48 hours. Next, PD-L1 surface presence was assessed by flow cytometry using anti-PD-L1 antibody (clone MIH1). Cytotoxic activity of PD-L1–CAR T cells against MCF-10A non-malignant cell line was measured by impedance analysis at the E:T ratios of 1:1 and 2:1. Samples were internally normalized for the cell index value measured before PD-L1–CAR T cells addition (Normalized Cell Index plots). The experiment was performed in duplicates three times. (C) PD-L1 expression induced on HMEC cells by activated CAR T cells (left panel) and RTCA-monitored cytotoxic activity of PD-L1 CAR T cells toward HMEC cells (right panel) was performed as described for (B). (D) PD-L1 expression induced on BM-MSC cells by activated CAR T cells (left panel) and cytotoxic activity of PD-L1–CAR T cells against BM-MSCs in the absence or presence of 25 ng/mL IFNγ was measured by impedance analysis at the E:T ratios of 1:1 and 2:1 (right panel). The experiment was performed in duplicates at least two times. (E) PD-L1 expression induced on HEK-293T cells by activated CAR T cells (left panel) and the killing potential of control (GFP-modified T cells) and PD-L1–CAR T cells against non-malignant human embryonic kidney cells HEK-293T pLVX (PD-L1^low/null^) and HEK-293T PD-L1 (PD-L1^+^) was determined by luciferase‐based killing assay following coculture of target and effector cells for 18 hours at different E:T ratios (right panel). The assay was repeated twice in triplicate and the results shown are representative of one experiment. CAR, chimeric antigen receptor; HMEC, human mammary epithelial cells, PD-L1, programmed death-ligand 1, IFNγ, interferon γ, GFP, green fluorescent protein, RTCA, real-time cell analysis.

To determine the cytotoxicity of PD-L1–CAR against normal cells, we have assessed PD-L1 protein presence in steady state in non-malignant mammary cell lines (spontaneously immortalized MCF-10A cell line and telomerase-immortalized human mammary epithelial cells (HMEC)), primary bone marrow-derived mesenchymal stromal cells (BM-MSC), and non-malignant embryonic kidney HEK-293T cell line. Both flow cytometry (figure 6A, left panels) and western blotting (figure 6A, right panels) demonstrated the low but detectable steady-state PD-L1 protein levels in MCF-10A and HMEC. For BM-MSCs, PD-L1 signal in western blotting was higher, but in flow cytometry, the surface PD-L1 expression remained below the level of detection. This indicates that in the steady state of BM-MSC, the PD-L1 molecule is located predominantly in the intracellular/cytoplasmic compartment(s). Lastly, no presence of PD-L1 protein was detected in HEK-293T cells by either of the abovementioned methods. Moreover, while MCF-10A, HMEC, and BM-MSC cells responded to the incubation with IFNγ by a significant increase in PD-L1 expression, HEK293T cells remained PD-L1 negative (figure 6A) on stimulation with IFNγ. Notably, the resistance of HEK293T cells to IFNγ-induced expression of PD-L1 is in accordance with the previous publication by Mimura *et al*.^15^ Finally, when all four cell types were incubated with the conditioned supernatants from CAR T cocultures with target MDA-MB-231 cells, the MCF-10A (figure 6B, left panel), HMEC (figure 6C, left panel), and BM-MSC (figure 6D, left panel) cells showed a spectacular increase in PD-L1 expression level, whereas the HEK293T cells, again, remained >99% PD-L1 negative (figure 6E, left panel).

### The activity of PD-L1–CAR effector cells against non-malignant cells

The surface presence of PD-L1, either in steady state or induced, on the majority of tested non-malignant cells suggested that these cells can also become targets to PD-L1–CAR-mediated cytotoxicity, either immediate or due to the self-amplification phenomenon described above. Indeed, both MCF-10A and HMEC cells were eliminated by PD-L1–CAR T cells in an E:T ratio-dependent manner, as presented in figure 6B, C (right panels), respectively. Moreover, when the BM-MSC cells were incubated with PD-L1–CAR T (RTCA assay, figure 6D, right panel) or PD-L1–CAR–NK-92 (fluorescent microscopy with the fluorescent probe detecting activation of caspase 3/7, online supplemental figure 7A), a potent cytotoxic effect was observed. Interestingly again, the steady-state BM-MSCs were eliminated by the PD-L1–CAR-bearing effector cells with latency as compared with the BM-MSC preincubated with IFNγ. This delay most probably results from the time-dependent induction of PD-L1 expression by the cytokines secreted by the CAR T cells. Simultaneously, the HEK293T cells, which were not prone to the cytokine-induced expression of PD-L1 (figure 6E, left panel), were almost completely resistant to the cytotoxic effects of PD-L1–CAR T cells, even in the high E:T (ie, 5:1) ratio (luminescence-based test, figure 6E, right panel, green bars). In order to exclude the possibility that these cells possess an impairment in response to the cytotoxic effect of immune effector cells, we have generated a PD-L1-overexpressing derivative of HEK293T cells (online supplemental figure 7B). These HEK293T–PD-L1 cells were readily killed by PD-L1–CAR T cells, while the empty-vector controls retained the resistance to cellular cytotoxicity (figure 6E, right panel, red bars), similar to the parental HEK293T cells. This substantiates the notion that the self-amplification effect of anti-PD-L1–CAR relies strictly on the capability of the target cells to increase PD-L1 expression in response to the cytokines released from the effector cells.

### Sequential killing by HER2–CAR T and PD-L1–CAR T combination

**Figure 7:**
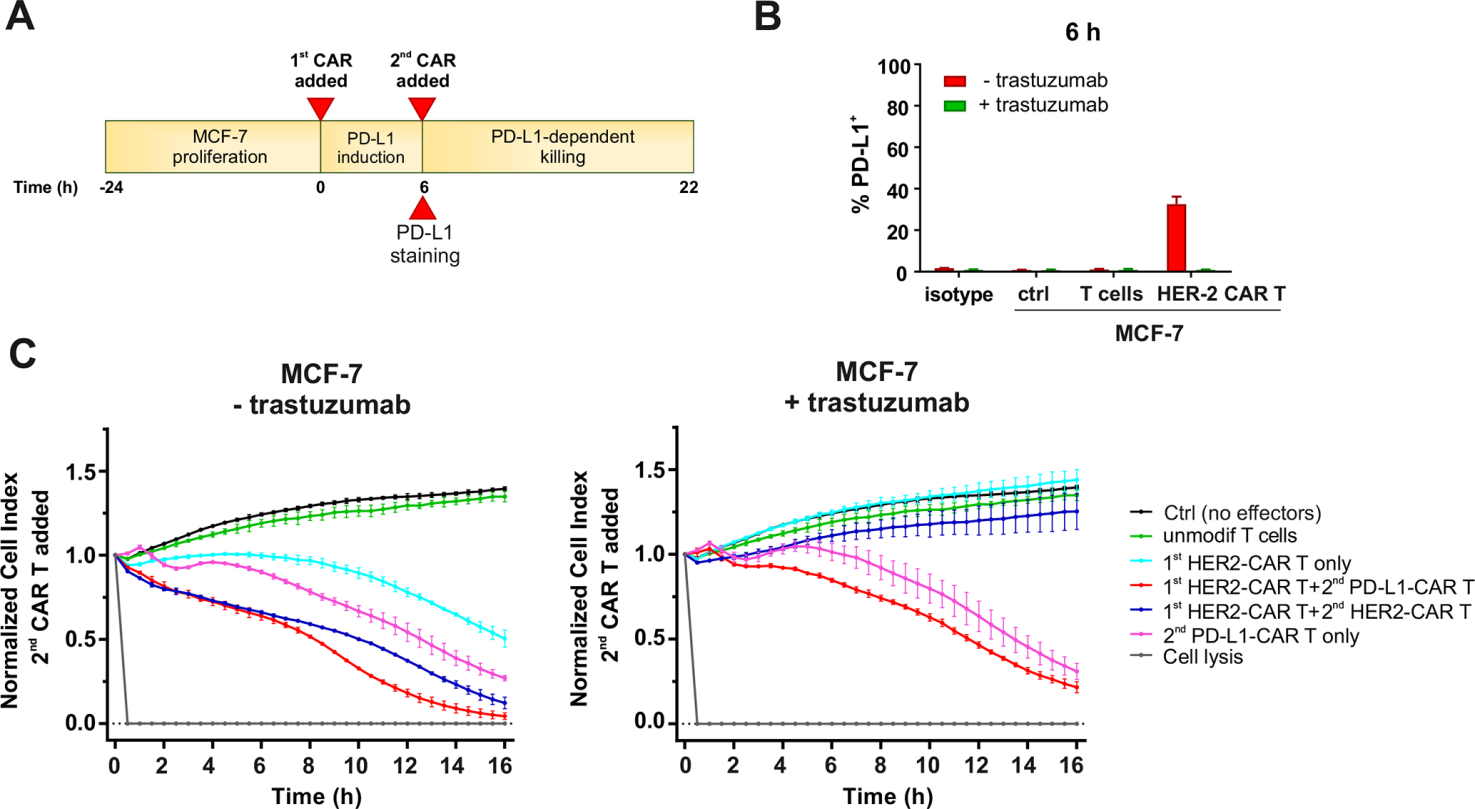
Sequential killing by HER2–CAR T and PD-L1–CAR T combination. (A) The schematic diagram of the sequential killing experiment. MCF-7 cells were left to proliferate for 24 hours. Next, MCF-7 cells were incubated with medium, HER-2–CAR or PD-L1–CAR T cells for 6 hours to induce PD-L1 expression on MCF-7 surface. Trastuzumab at the concentration 100 µg/mL was used as a blocking antibody for HER-2 antigen. After 6 hours, a fresh portion of the medium, PD-L1–CAR or HER-2–CAR T cells were added, followed by measuring the killing potential for the next 16 hours. Arrow indicates the time of surface staining of PD-L1 by flow cytometry performed in the parallel experiment (B). (B) PD-L1 expression induced on MCF-7 cells after 6 hours of coincubation with HER-2–CAR T cells in presence or absence of 100 µg/mL trastuzumab. PD-L1 surface presence was assessed using an anti-PD-L1 antibody (clone MIH1) by flow cytometry. The experiment was repeated in duplicates two times. (C) The sequential killing of MCF-7 cell lines was measured by impedance analysis in RTCA assay. CAR T cells were added at the E:T ratio of 0.5:1 with or without 100 µg/mL trastuzumab supplementation. Representative mean impedance curves from two wells after the addition of the second portion of PD-L1–CAR T cells are shown. The experiment was repeated in duplicates at least two times. CAR, chimeric antigen receptor, PD-L1, programmed death-ligand 1, HER2, human epidermal growth factor receptor 2, RTCA, real-time cell analysis.

Having identified the phenomenon of PD-L1 induction in target cells following the interaction with PD-L1–CAR effector cells, we have decided to interrogate the strategy to increase the precision of PD-L1–CAR-based therapy and the susceptibility of the tumor to this CAR. Specifically, the results described above (figure 5D) brought us to the conclusion that solid-tumor targeted CAR T cells (eg, HER-2–CAR T cells) can ‘prepare’ (ie, sensitize by increasing PD-L1 expression) cancer cells and TME to the actions of PD-L1–CAR T cells. To test this hypothesis, we carried out sequential, combined incubations of MCF-7 cells with HER-2–CAR T and/or PD-L1–CAR T cells (please refer to figure 7A for the experiment schematic). Notably, we have used suboptimal, regarding the cytotoxic activity (online supplemental figure 6C), E:T ratio of HER-2–CAR T cells in order to avoid the high background cytotoxicity. Importantly, HER-2–CAR T cells in such E:T ratio successfully induced PD-L1 expression in a considerable percentage of target MCF-7 cells within 6 hours (figure 7B, red bars), marking these cells for PD-L1–CAR T targeting. Indeed, we observed that target cells preincubated with HER-2–CAR T cells responded to PD-L1–CAR T cells without delay, while when PD-L1–CAR T cells were added to cells without prior HER-2–CAR T treatment, the 4-6-hour delay in cytotoxic response was present again (figure 7C, left-hand panel). This strongly indicates the validity of such an approach. Moreover, the delay in cytotoxicity of PD-L1–CAR T cells against MCF-7 cells was retained when the experiment was carried in the presence of trastuzumab (figure 7C, right panel), that is, the antibody competitively blocking the binding of HER-2–CAR to its target and subsequent induction of PD-L1 (figure 7B, green bars). From these experiments, we conclude that PD-L1–CAR can play a supportive role for other solid tumor-targeted CARs by eliminating the reactive PD-L1-positive cells, if applied sequentially after the tumor-specific CAR, even in a low E:T ratio. In such settings, PD-L1–CAR T cells would eradicate cancer cells, but additionally may also kill the TME cells (such as tumor-associated macrophages), if those were responding to the first CAR-based treatment by upregulating PD-L1 immune checkpoint on their surface. It should be noted, that in such an approach, the HER-2–CAR T cells would be also eliminated by sequentially added PD-L1–CAR T cells, but this phenomenon can, in theory, be avoided by knocking out (eg, by CRISPR/Cas9 approach) *CD274* gene in HER2–CAR T cells. This subject warrants further investigations.

## Discussion

PD-L1 immune checkpoint molecule is an attractive target for the immunotherapeutic strategies against a range of human malignancies, with a special emphasis on solid tumors.^2 16^ This is due to the fact that not only PD-L1 molecule, expressed on a significant number of cancer cells, but also other cells within the TME, are documented to inhibit the antitumor function of immune effector cells. An obvious advantage of the CAR-based approach over the inhibition of the PD-L1/PD-1 axis by the most of the anti-PD-L1 monoclonal antibodies^17^ is a permanent effect of the physical elimination of the PD-L1-expressing target cells within TME. Therefore, it can be expected that PD-L1–CAR cells could not only be used for killing the PD-L1^high^ malignant cells per se but may also induce the reshaping of TME by eradication of its immunosuppressive PD-L1-positive cellular components. The same holds true for the CAR (other than anti-PD-L1 CAR) T cells plus anti-PD-L1 antibody combination approach in the solid tumors, although the activity of CAR T cells against the malignant cells can indeed be increased by the anti-PD-L1 antibodies.^18^

However, an important question is whether the PD-L1^low/null^ tumors should be disregarded for the PD-L1–CAR T-based/NK-based treatment, as initially suggested.^19^ We addressed this question in our study by investigating the responsiveness of initially PD-L1^low/null^ targets to the PD-L1–CAR-mediated cytotoxicity. In this study, we describe a potent and time-dependent ability of activated immune effector cells bearing PD-L1–CAR to induce the expression of PD-L1 molecule on the surface of a number of cell types, either in a juxtracrine or paracrine manner. This can make the initially PD-L1^low^ cells, such as MCF-7 cell line, vulnerable to the PD-L1–CAR-mediated cytotoxicity. In this context, our results are in accordance with the work by Robbins *et al*, which demonstrated that the effectiveness of PD-L1–CAR-bearing effector cells can be significantly amplified following the preincubation of cancer cells with IFNγ.^7^

Moreover, it has already been widely documented by others that the expression of PD-L1 can be potently induced by various proinflammatory agents, such as TNFα or IFNγ (reviewed by Ribas)^2^ or direct contact with immune effector cells.^20^ Hereby, we report a massive induction of PD-L1 as a consequence of antigen-specific CAR activation and production of cytokines. Activated immune effector cells are well-known sources of a broad set of proinflammatory factors, which we show in the current work to synergistically upregulate surface PD-L1 expression to the highest levels, as compared with IFNγ only treatment (figure 5D). Similar to our observations, trastuzumab (anti-HER2 antibody) was shown to upregulate PD-L1 level in HER2-overexpressing human breast cancer cells by stimulating human peripheral blood mononuclear cells to release IFNγ.^21^

Importantly, the effect of amplifying the expression of PD-L1 on the target or bystander cells by the activated CAR-bearing immune effector cells can have both positive and negative consequences. We consider this self-amplification phenomenon, exceptional in comparison with other CAR-based therapies, to be vital for further studies on this CAR, as it may significantly broaden the spectrum of potential targets for the PD-L1–CAR-based strategies. Our results obtained from the experiments with macrophages (figure 5B), a classical constituent of TME, suggest a potential paracrine effect of target-stimulated CAR T cells. This would additionally broaden the cytotoxic reaction of PD-L1-CAR cells toward the tumor stroma cells adjacent to the malignant cells, even if the stromal cells were initially PD-L1^low/null^.

The PD-L1 amplification phenomenon by the activated CAR effector cells may have a broader effect and influence the efficacy of various CAR-based approaches. Given the role of PD-L1 molecule as a negative regulator of the T-cell immune response, upregulation of PD-L1 following the treatment with CAR T cells may function as a mechanism of resistance to CAR-based strategies employed against solid tumors. This finding encourages further investigation of the advantage of adding anti-PD-1/PD-L1 therapy to CAR-based treatments (reviewed by Kosti *et al)*^22^, especially in patients with low constitutive PD-L1 expression. Interestingly, the combination of high-affinity PD-L1-CAR NK cells withthe IL-15 superagonist and anti-PD-1 antibody has been already demonstrated by others to have superior tumor growth control of engrafted oral cavity squamous carcinoma tumors in mice.^23^ The effect of amplifying the PD-L1 expression can also serve as a stimulus for incorporating the PD-L1–CAR into combinations with other CAR-based strategies. Indeed, in this work, we provide a proof of concept for sensitizing the breast cancer cells to PD-L1–CAR T cells by their prior incubation with HER-2–CAR T cells (figure 7). Therefore, our data suggest that due to the effect of reactive expression of PD-L1 on target cells after their incubation with CAR T cells, the PD-L1–CAR T cells may be used to amplify the effectiveness of alternative CARs applied in solid tumors treatment. Surprisingly, however, when PD-L1–CAR T cells were used in addition to the mesothelin-targeting CAR T cells in the previous work by Qin *et al*,^24^ an antagonistic interaction was observed. The explanation relies on the fact that PD-L1–CAR T cells can induce fratricide effects against other activated CAR T cells, as the latter start expressing PD-L1 on their surface following recognition of the target. Indeed, we report a similar phenomenon in this work, that is, the activated CD19–CAR T cells readily expressed PD-L1 on their surface, while the PD-L1–CAR T cells remained PD-L1^null^ following activation (online supplemental figure 3B), which might be attributed to masking PD-L1 by PD-L1–CAR on the cell surface, as suggested previously,^24^ or, alternatively, elimination of the T-cell subpopulation with inducible PD-L1 expression by those PD-L1–CAR T cells, which do not express PD-L1. We respond to this question in our study, by presenting that PD-L1–CAR T cells lose the capability of expressing PD-L1 protein (online supplemental figure 3D); however, they retain the expression of PD-L1 mRNA on induction by activatory stimulus (online supplemental figure 3E). The exact mechanism of this phenomenon warrants further investigations.

These observations imply that in combinatory approaches involving PD-L1–CAR T cells, the expression of PD-L1 on other CAR T cells should be prevented, for example, by genetic KO of the *CD274* gene, RNAi-mediated knockdown, or in vitro preincubation of other CAR T cells with PD-L1–CAR cells. An additional benefit from suppressing the PD-L1 molecule in CAR T cells can be related to the fact that PD-L1 engagement on T cells has been shown to promote the suppression of neighboring macrophages and effector T cells in cancer.^24^ Moreover, PD-L1 expression on T cells has been very recently demonstrated to play an important role in the suppression of T cells, and PD-L1^+^ T cells have diverse tolerogenic effects on tumor immunity.^25^ Thus, removing the ability of PD-L1 expression from the CAR T cells would not only protect them from PD-L1–CAR T cells used in combination, but also might amplify their effectiveness per se. This issue warrants further investigation.

The negative consequences of the PD-L1 amplification phenomenon are related to the safety issues of this strategy in clinical settings. PD-L1 molecule is involved in peripheral immune self-tolerance toward numerous healthy tissues, with the highest steady-state expression level not only in the placenta but also in vital organs such as lungs, intestine, or heart. Accordingly, the risk of inducing the immune-related adverse effects by tampering with the PD-L1 functions is clearly demonstrated in patients treated with anti-PD-L1 checkpoint blockers (reviewed by Choi and Lee).^26^ Therefore, it is of the utmost importance to properly assess the potential toxicities of PD-L1–CAR-based strategies against the non-malignant cells. In our work, we have used four non-malignant types of cells (both established cell lines and primary cells) to investigate the toxicity of PD-L1–CAR T/NK-92 cells. Our data show that PD-L1–CAR T cells can exhibit potent cytotoxicity against non-malignant cells, as long as those express PD-L1 molecule in steady state (MCF-10A and HMEC cells) or in response to the proinflammatory stimulus (BM-MSC). The only cell line resistant to the PD-L1–CAR-mediated toxicity was HEK293T, which was PD-L1^null^ initially and remained refractory to the induction of PD-L1 expression either by IFNγ or the conditioned medium derived from cultures of activated CAR T cells. Importantly, the exogenous expression of PD-L1 on HEK293T cells made them sensitive to the PD-L1–CAR T-mediated toxicity, eliminating the possibility that these cells were resistant to cell-mediated cytotoxicity in general.

The sensitivity of non-malignant cells to PD-L1–CAR-mediated toxicity suggests that PD-L1–CAR-based therapies should be evaluated with extreme caution while applied in humans. This notion is especially highlighted by the fact that the clinical trial in patients with advanced lung cancer involving PD-L1–CAR T cells (ClinicalTrials.gov Identifier: NCT03330834) was recently terminated due to serious, however, resolved on tocilizumab and steroids treatment, adverse effects after one patient received the treatment. In this regard, previous groups testing PD-L1–CAR T cells in human-to-mouse xenotransplantation tumor models reported no apparent generalized toxicity of the therapy in mice, even though atezolizumab-based CARs are expected to be cross-reactive with mouse PD-L1. It must be underscored, however, that human T cells can induce immense graft-versus-host reaction in the mouse body,^27^ which can significantly mask the delayed toxicity of PD-L1–CAR. Therefore, the preclinical safety studies with PD-L1–CAR must be appropriately designed in order to identify the potentially severe on-target, off-tumor adverse effects. In the case of detecting the generalized toxicity of PD-L1–CAR-based therapies, further studies must be conducted in order to optimize the precision of these strategies toward malignant burden and minimize the risk of the toxicities to the healthy tissues. Theoretically, that might be achieved by controlled/inducible expression of PD-L1–CAR, such as hypoxia-sensing system,^28^ transient expression by mRNA electroporation, or application of agents neutralizing proinflammatory factors^21^ and thus preventing the PD-L1 self-amplification effect. Also, it seems plausible that since NK cells are not so abundantly producing proinflammatory cytokines, modifications of NK cells and NK-92 cell line, instead of T cells, would be a more appropriate design for PD-L1–CAR to avoid the rolling snowball effect and off-tumor toxicity. Indeed, PD-L1–CAR ha (high affinity) NK cells were already shown by others to be effective and safe in the treatment of immunodeficient mice bearing human head and neck cancer xenograft tumors,^7^ as well as triple-negative breast cancer (TNBC), lung or bladder tumors.^23^ However, the results presented in current work, as presented in figure 5C, indicate that the pattern of cytokines produced by target-activated CAR–NK-92 can be still sufficient for induction of PD-L1 on the target and bystander cells. This subject requires further assessment.

In summary, we demonstrate the efficacy of PD-L1–CAR T/NK/NK-92 cells against a model of triple-negative breast cancer intrinsically expressing high levels of PD-L1.^29^ Our work corroborates the notion that PD-L1 is an attractive target for prospective CAR-based anticancer therapies and could be applied in a considerable percentage of malignancies of various histopathological types to directly eliminate tumor cells and TME cells expressing PD-L1 on their surface.

This study also brings new information on the potential application of PD-L1 CAR against PD-L1^low/null^ tumors, as long as they respond to proinflammatory cytokines by upregulation of PD-L1 on the cellular surface. Also, our results imply that targeting PD-L1 raises the considerable risk of on-target, off-tumor reactivity of the PD-L1–CAR-bearing effector cells toward non-malignant tissue and help to explain the mechanisms of potential toxicities of PD-L1–CAR T cells. We suggest the need to find safer approaches using the PD-L1–CAR-bearing effector cells and propose an example strategy for PD-L1–CAR as an agent supporting and amplifying the effectiveness of other CARs when administered sequentially. Collectively, based on our observations, we assume that the potential targets for CAR-based therapies should be regularly analyzed for their inducibility by proinflammatory cytokines, as this may change the specificity and safety spectrum and finally change the validity of a given target. We believe that the information presented in this study may change the future approaches aimed at the development of successful CAR-based therapies against solid tumors.

## Data Availability

Data sharing not applicable as no datasets were generated and/or analyzed for this study. All data relevant to the study are included in the article or uploaded as supplementary information.
