# Correction: PD-L1 CAR effector cells induce self-amplifying cytotoxic effects against target cells

**DOI:** 10.1136/jitc-2021-002500corr1

**Published:** 2022-03-09

Bajor M, Graczyk-Jarzynka A, Marhelava K, *et al*. PD-L1 CAR effector cells induce self-amplifying cytotoxic effects against target cells. *J ImmunoTher Cancer* 2022;10:e002500. doi: 10.1136/jitc-2021-002500

The funding statement for this paper was incorrect. The funding statement has been updated to:

The work was supported by the grants from the National Science Centre, Poland (2016/23/B/NZ6/02535, RZ; 2019/35/D/NZ6/03073, AG-J, and 2019/33/B/NZ6/02503, MB), the Polpharma Scientific Foundation (5FPOL8, AG-J), the European Research Council (805038/STIMUNO/ERC-2018-STG, MW), the Medical Research Agency (2020/ABM/04/00002), and the Ministry of Science and Higher Education within ‘Regional Initiative of Excellence’ (013/RID/2018/19, 2019–2022).

