# Supplementary material for: PD-L1 CAR effector cells induce self-amplifying cytotoxic effects against target cells
Source: J Immunother Cancer. 2022 Jan 24;10(1):e002500. doi: 10.1136/jitc-2021-002500 (PMC8796262; doi:10.1136/jitc-2021-002500)
Supplement: Supplementary data [file jitc-2021-002500supp001.pdf]

Supplementary data.

Materials and methods.

Cell lines

Human breast carcinoma cell lines: MCF-7 (RRID:CVCL_0031), ZR-75-1 (RRID:CVCL_0588), T47D (RRID:CVCL_0553), MDA-MB-231 (RRID:CVCL_0062), HCC-1806 (RRID:CVCL_1258), SKBR-3 (RRID:CVCL_0033), and a non-malignant immortalized mammary cell line MCF-10A (RRID:CVCL_0598) were purchased from the European Collection of Cell Cultures (Wiltshire, UK). Cells were cultured in DMEM (ZR-75-1, T47D) or RPMI-1640 (MCF-7, MDA-MB-231, HCC-1806, and SKBR-3) media (Sigma-Aldrich, St Louis, MO, USA) supplemented with 10% fetal bovine serum (FBS) (Sigma-Aldrich), 2 mM L-glutamine (Sigma-Aldrich) and 1% antibiotics – penicillin/streptomycin (Sigma-Aldrich) in a humidified atmosphere containing 5% CO_2. Additionally, ZR-75-1 and T47D culture media were supplemented with 1nM β-estradiol (Sigma Aldrich). MCF-10A was cultured in mammary epithelial basal media (MEBM, Lonza) containing 0.4% bovine pituitary extract (BPE), 10 ng/ml human epidermal growth factor (hEGF), 5 µg/ml human insulin, 0.5 µg/ml hydrocortisone, 30 µg/ml gentamicin and 15 µg/ml amphotericin, and 100 ng/ml cholera toxin (Sigma-Aldrich). HMEC, primary human mammary epithelial cells, were purchased from Life Technologies (Carlsbad, CA). HMEC cells were cultured in HuMEC medium supplemented with epidermal growth factor, hydrocortisone, isoproterenol, transferrin, insulin, and 50 µg/ml bovine pituitary extract, according to manufacturer protocol (Life Technologies). HEK-293T (RRID:CVCL_0063) and Phoenix Ampho (RRID:CVCL_H716) cells were purchased from ATCC and were cultured in DMEM medium supplemented with 10% FBS. NK-92 cell line (RRID:CVCL_2142, a generous gift from Prof. Kerry Campbell) was maintained in X-VIVO™ 20 medium (Lonza) supplemented with 5% human serum (Sigma Aldrich). All the cell lines were authenticated in Eurofins Genomics, maintained through continuous passaging, and were confirmed to be free of contamination with Mycoplasma spp.

Peripheral blood mononuclear cells (PBMCs) and T cells selection

Peripheral blood mononuclear cells (PBMCs) were isolated from the whole blood by density gradient separation using Histopaque-1077 (Sigma-Aldrich, St Louis, MA, USA). The procedure was approved by the Local Bioethics Committee (approval number: AKBE/184/2018). After gradient centrifugation, PBMCs were seeded onto a 6-well plate at a cell density 1×10^6 per 1 ml in 3 ml RPMI-1640 medium supplemented with 10% FBS and 1% penicillin and streptomycin solution and stimulated using 2.4 µg/ml of phytohemagglutinin-L (PHA-L, Sigma Aldrich) for 3 days. After transduction, T cells were maintained in full RPMI-1640 medium supplemented with 100 U/ml IL-2 (Peprotech) and in the

presence of Dynabeads Human T-Activator CD3/CD28 (Thermo Fisher Scientific) at a bead-to-cell ratio of 1:5.

Macrophage isolation and differentiation

Freshly isolated PBMCs were suspended in RPMI-1640 (Biowest) and seeded onto 96-well, µCLEAR, black plates (Grainer, Ref No. 655090) at a cell density 3×10^5 per well in 100 µl. After 2 h of incubation in standard conditions (37°C, 5% CO_2, 95% humidity), the cells were washed once with PBS to remove unattached cells. Afterward, the remaining adherent cells considered as monocytes-enriched PBMCs were cultivated in the medium consisting of RPMI-1640 supplemented with 10% human serum (Sigma-Aldrich) and antibiotics (1% Penicillin-Streptomycin) in standard conditions. On the 3rd day of culture, the medium was changed, and the monocytes/macrophages either remained untreated or were stimulated with IFNγ (10 ng/ml) or a combination of IL-4 and IL-10 (10 ng/ml both). A fresh portion of the cytokines was added every other day.

Bone marrow collection

The bone marrow samples were obtained from patients after receiving their written consent. The procedure was approved by the Local Bioethics Committee (approval number: KB/115/2016). The bone marrow was collected during standard orthopedic surgeries, which required the opening of the bone marrow cavity.

Mesenchymal stromal cells isolation

Isolation was performed on the same day as the bone marrow was collected. The bone marrow, after mechanical disassociation, was washed with PBS and centrifuged at 300 × *g* for 5 min. The samples were then expanded in DMEM-LG growth medium (Dulbecco's modified Eagle's Medium with low glucose; Lonza) supplemented with 10% fetal calf serum (FCS, Lonza) and 1.5% antibiotic–antimycotic (AA) solution (Penicillin-streptomycin-amphotericin B, Invitrogen), seeded on BD Primaria™ culture dishes (Becton Dickinson) and incubated under standard cell culture conditions (37°C, 5% CO_2, 95% humidity, and atmospheric oxygen concentration). After 4 days, non-adherent cells were removed, and first fibroblast-like adherent cells could be observed. When the colonies were well-formed (usually 7-10 days after isolation), the cells were passaged. Subsequent passages were performed when cells were reaching about 80% of confluence. After the third passage, the cells were frozen in liquid nitrogen. After thawing, cells underwent an identification process, which included differentiation assays and the analysis of cell surface antigen profile. The experiments were performed on the cells between 4 to 5 passage. The cells from different donors were always cultured separately.

Immunocytochemistry (ICC) analysis of PD-L1 and CD206 in macrophages

PD-L1 and CD206 expression in macrophages was assessed after 4 days of differentiation. T cells or PD-L1-CAR T cells conditioned supernatants from the 24-hour co-incubation with MDA-MB-231 cell line were placed instead of the medium for 48 h to assess the impact of these supernatants on induced PD-L1 expression. The cells were fixed with 4% paraformaldehyde (15 min, at 25°C). After washing, the cells were incubated with blocking buffer consisting of PBS with 2.5% normal donkey serum and 1% bovine serum albumin for 30 min (at 25°C), followed by overnight incubation (at 4°C) with primary goat anti-CD206 antibody (R&D Systems Cat# AF2534, RRID:AB_2063019, dilution 1:100) and primary mouse anti-PD-L1 antibody (Thermo Fisher Scientific Cat# 14-5983-82, RRID:AB_467784, dilution 1:100) solution in blocking buffer. Next, after washing, the cells were incubated with secondary antibodies (Alexa Fluor 647 donkey anti-goat IgG (H+L), Molecular Probes Cat# A-21447, RRID:AB_141844 and Alexa Fluor 488 donkey anti-mouse IgG (H+L) Molecular Probes Cat# A-21202, RRID:AB_141607) in blocking buffer (both 1:300) for 1 h at 25°C. Nuclei were stained with DAPI solution (20 ng/ml, for 4 min, at 25°C). The cells were visualized using automated imaging reader Cytation™ 1 (BioTek, Agilent). The images were analyzed using Gen5 3.04 software (BioTek, RRID:SCR_017317). The mean fluorescence intensity within secondary masks was measured. The secondary mask was designed around each DAPI stained nucleus (which constituted a primary mask).

Western blotting

Total protein lysates were prepared in the lysis buffer (50 mM Hepes, 150 mM NaCl, 5 mM EDTA, 1.0% (w/v) Triton X-100, 10% (v/v) glycerol, pH 7.4) supplemented with Complete Protease Inhibitor Cocktail (Roche Diagnostics, Basel, Switzerland). Protein concentration was determined using Pierce BCA Protein Assay Kit (ThermoFisher Scientific, Waltham, MA, USA) according to the manufacturer's instructions. Equal amounts of total protein lysates (20 µg) were separated in 12% (v/v) reducing SDS-polyacrylamide gel, then transferred onto nitrocellulose membranes, and blocked with 5% (w/v) nonfat dry milk in TBST (Tris-buffered saline, pH 7.4 and 0.05% (v/v) Tween-20) for 1 h at 25°C. Afterward, the membrane was incubated overnight at 4°C with the following primary antibodies: anti-PD-L1 (dilution 1:1000, Cell Signaling Technology Cat# 13684, RRID:AB_2687655) and anti-β-actin-HRP (1:40000 dilution, Sigma-Aldrich Cat# A3854, RRID:AB_262011). For the detection of specific protein bands, HRP-conjugated secondary antibodies were used. The blots were exposed to the Super Signal chemiluminescent substrate (Thermo Fisher Sci. Rockford, USA) and detected using ChemiDoc Imaging System (Bio-Rad ChemiDoc MP Imaging System, RRID:SCR_019037).

RNA isolation and quantitative real time PCR

Total RNA was isolated using RNeasy kit (Qiagen). RNA concentration was measured with NanoDrop 2000 spectrophotometer (Thermo Fischer Scientific, Waltham, MA, USA) and 500 ng of RNA was

reverse transcribed to complementary cDNA with the use of Superscript IV Reverse Transciptase kit and random hexamer primer (Thermo Fischer Scientific, Waltham, MA, USA) according to the manufacturer's instructions. Quantitative real-time PCR (qPCR) with LightCycler 480 SYBR Green I Master (Roche Diagnostics, Basel, Switzerland) was performed using LightCycler 480 II device (Roche Diagnostics, Basel, Switzerland) according to the manufacturer's recommendations in 20 µl final volume. In each PCR run, the samples were measured in duplicates. Primers used within the study are listed in the tble below:

F-PD-L1	5'-GCT GAA TTG GTC ATC CCA GAA-3'
R-PD-L1	5'-TTT CAC ATC CAT CAT TCT CCC TT-3'
F-SDHA	5'-GCA TTT GGC CTT TCT GAG GC-3'
R-SDHA	5'-CTC CAT GTT CCC CAG AGC AG-3'
F-TBP	5'-GCA CAG GAG CCA AGA GTG A-3'
R-TBP	5'-GTT GGT GGG TGA GCA CAA G-3'

Fluorescence Activated Cell Sorting

NK-92 cells from 80% confluent culture flasks were centrifuged and resuspended in PBS in a final concentration of 5 × 10^6 cells/ml. Then, the cells were transferred through a cell strainer to ensure separation of the cells. The sorting was performed according to surface CAR expression with FACS Aria III cell sorter (BD Biosciences, La Jolla, CA, USA).

CAR construction

PD-L1-targeting CAR synthesis was performed by Creative Biolabs, and the design was as follows: atezolizumab-based scFv sequence was combined with the CAR backbone consisting of full IgG4 hinge, CD28 transmembrane region, CD28, and CD3ζ signaling domains. Next, it was sub-cloned into the BamHI/SbfI restriction sites of the lentiviral transfer plasmid pSEW [1], thereby replacing the *gfp* gene. For some experiments (in vivo settings) atezolizumab based scFv was subcloned to CAR backbone with half IgG1 hinge, CD28 transmembrane region, CD28, and CD3ζ signaling domains and insereted into pSEW vector. HER2-CAR construct was designed from trastuzumab sequence (4D5, https://www.drugbank.ca/drugs/DB00072). Briefly, a degenerated DNA coding sequence of the scFv was ordered to Eurofins-MWG (Ebersberg, Germany) with a "light chain-$(G_3S)_3$ linker-heavy chain" structure as in [2]. The sequence was inserted into a second-generation CAR-backbone containing a CD8 hinge and transmembrane domain and a 4-1BB-CD3ζ signaling tail, as described in [3] and also, subsequentely, subcloned to pSEW lentiviral plasmid. For HER-2-CAR detection, a sequence coding a

truncated form of CD34 was inserted into the vector. Two CD19-CAR (FMC63 clone) constructs were used in this study, both, a kind gift from M. Pule (UCL, UK). SFG retroviral plasmid comprises the CD8 hinge and transmembrane domain, 41BB costimulatory domain, CD3ζ signaling domain, and rituximab recognized-RQR8 epitope for CAR detection. The second CD19-CAR construct comprises IgG1 half-hinge, CD28 transmembrane and co-stimulatory domain and CD3ζ and was inserted into pSEW plasmid.

Lentiviral and retroviral T cells modifications

PBMC isolated from buffy coats were seeded onto the 6-well plate at a cell density 1 × 10^6 cells per 1 ml in 3 ml RPMI full medium and stimulated using 2.4 ug/ml of PHA-L for 3 days. To produce control or CAR viral particles, HEK-293T cells were seeded at 10 cm plates – 4 plates per virus and transfected using a polyethyleneimine (PEI) transfection protocol to deliver a transfer plasmid with gene of interest simultaneously, VSV-G envelope expressing plasmid pMD2.G (a gift from Didier Trono; RRID:Addgene_12259) and lentiviral packaging plasmid psPAX2 (a gift from Didier Trono; RRID:Addgene_12260). After 48 h, the lentivirus-containing supernatant was harvested, filtered through a 0.45 µm pore size filter, and concentrated by overnight centrifugation at 2 500 × *g* at 4°C. The culture medium from the stimulated PMBC was replaced with concentrated lentiviral supernatant, and 4 µg/ml of polybrene (Sigma-Aldrich) was added. After 1 h of spinoculation round (2 000 × *g* at 32°C), the PMBC were kept in a humidified atmosphere containing 5% carbon dioxide (CO_2). The next day, the viral supernatant was replaced with the fresh portion of complete culture RPMI-1640 medium supplemented with 200 U/ml IL-2 (PeproTech) and Dynabeads Human T-Activator CD3/CD28 (Thermo Fisher Scientific) at a bead-to-cell ratio of 1:5). In the case of retroviral transduction, 2 x 10 cm plates of Phoenix Ampho cells were seeded per virus, and a gene of interest was co-transfected together with pHIT60 and pCOLT-GALV plasmids [4], a kind gift of Tuna Mutis (VUmc the Netherlands). Retroviral supernatant was collected, spun down at 500 x g for 5 min, and concentrated using Retro-X-Concentrator (Takara). Stimulated PBMC's were transferred on the 50 µg/ml Retronectin (Takara) coated plates, and concentrated retroviral supernatant was applied. After 1 h of spinoculation round (2 000 × *g* at 25°C), the PMBC were kept in a humidified atmosphere containing 5% carbon dioxide (CO_2). The spinoculation with the second batch of viral supernatant was repeated on the next day. Similar to lentiviral transduction, on the following day, the viral supernatant was replaced with the fresh portion of complete culture RPMI-1640 medium supplemented with 200 U/ml IL-2 (PeproTech) and Dynabeads Human T-Activator CD3/CD28 (Thermo Fisher Scientific) at a bead-to-cell ratio of 1:5. The CAR expression on the surface of the T cells was evaluated by flow cytometry 48-72 h after transduction.

Lentiviral modification of NK-92 cells

To produce control or PD-L1-CAR viral particles, HEK-293T cells were seeded at 10 cm plates – 4 plates per virus and transfected using a polyethyleneimine (PEI) transfection protocol to deliver a transfer plasmid with gene of interest simultaneously, VSV-G envelope expressing plasmid pMD2.G (RRID:Addgene_12259) and lentiviral packaging plasmid psPAX2 (RRID:Addgene_12260). After 48 h, the lentivirus-containing supernatant was harvested, filtered through a 0.45 µm pore size filter, and concentrated by overnight centrifugation at 2 500 × *g* at 4°C. The culture medium from the NK-92 cells was replaced with concentrated lentiviral supernatant supplemented with 15 µg/ml protamine sulfate and 6 µM BX-795. After 1 h of spinoculation (750 × *g* at 25°C), the NK-92 cells were kept in a humidified atmosphere containing 5% carbon dioxide (CO_2). The next day, the viral supernatant was replaced with the fresh portion of complete culture X-VIVO™ 20 medium (Lonza) supplemented with 200 U/ml IL-2 (PeproTech). The CAR expression on the surface of the NK-92 cells was evaluated by flow cytometry 48-72 h after transduction.

In vitro mRNA synthesis and mRNA electroporation

For CAR mRNA synthesis *in vitro,* the PD-L1-CAR construct was subcloned into pCIpA102 plasmid (a kind gift of Stein Sæbøe-Larssen from OUS). Linearized plasmid was cleaned up using QIAquick PCR Purification Kit (Qiagen) and was utilized as a template in mRNA synthesis reaction (Promega RiboMax Large Scale). ARCA (Anti Reverse Cap Analog) was used in the mRNA synthesis reaction to ensure proper cap orientation and by that 100% mRNA activity. Produced mRNA in concentration 1 µg/µl was stored at -80°C. Primary NK cells were magnetically isolated from PBMC using EasySep™ Human NK Cell Isolation Kit (StemCell Technologies) and activated for 3 days by adding 20 ng/ml IL-15. Then, the cells were electroporated with PD-L1-CAR mRNA. The procedure was performed using Gene Pulser Xcell Electroporation Systems (BioRad) in 0.4 cm Gene Pulser/Micropulser electroporation cuvettes (BioRad). 2×10^6 of NK cells suspended in 400 µl of Opti-MEM Reduced Serum Medium (ThermoFisher) were mixed with 20 or 40 µl (1 µg/µl) of previously synthesized mRNA or water (for MOCK control). After the electroporation (500 V, 2 ms, squarewave), cells were transferred onto a 6-well plate containing 2 ml of pre-warmed complete medium supplemented with 20 ng/ml of IL-15 for NK cells. After 18 h, the PD-L1-CAR expression was evaluated by flow cytometry.

CAR surface staining

To assess CAR surface expression, the effector cells (T, NK, or NK-92 cells) were washed 3 times with EasySep buffer (PBS supplemented with 2% FBS and 1 mM EDTA) and stained using goat anti-human IgG, Fcγ fragment specific antibody conjugated with Alexa Fluor 647 (Jackson ImmunoResearch Labs Cat# 109-606-098, RRID:AB_2337899, dilution 1:200) detecting a CH2-CH3 full hinge or CH3 half hinge region of the PD-L1-CAR or CD19-CAR. For cells modified with SFG CD19-CAR construct rituximab was

used at the concentration 10 µg/ml with consecutive anti-human IgG, Fcγ fragment specific antibody conjugated with Alexa Fluor 647. For HER2-CAR detection, the truncated version of human CD34 was detected by CD34 monoclonal antibody conjugated with APC (Thermo Fisher Scientific Cat# 17-0349-42, RRID:AB_2016672, dilution 1:50).

Generation target cell lines for luciferase based killing assays

HEK293T cells were modified with plasmid pLenti7.3/V5 TOPO-RedLuc encoding the red luciferase gene and green fluorescent protein, as described previously [5].

Generation of PD-L1- knockout and PD-L1-overexpressing target cell lines

Target cell lines unmodified or expressing luciferase reporter were used to generate cell models varying in PD-L1 expression. To obtain PD-L1 knockout of MDA-MB-231 cells, sgRNA sequences towards PD-L1 were selected from the Human CRISPR Knockout Pooled Library Brunello database (ACATGTCAGTTCATGTTCAG and ACTGCTTGTCCAGATGACTT) and cloned into the lentiviral vector pLentiCRISPv.2 plasmid, which was a gift from Feng Zhang (RRID:Addgene_52961 [6]). As a control, the construct with sgRNA towards a non-mammalian targeting control (NTC) was used. To generate MCF-7 and HEK-293T-PD-L1 overexpressing cells, the cDNA sequence of PD-L1 (NM_014143.2, Origene) was cloned into pLVX-IRES-PURO. MCF-7 and HEK-293T cells were modified with pLVX-IRES-PURO or pLVX-PD-L1-IRES-PURO constructs using lentiviral transduction. Two days after transduction, puromycin at 2 µg/ml was added to enforce the selection of the modified cells.

Induction of PD-L1 on the cell surface of effector cells

PD-L1 expression on unmodified T cells or CAR-modified T cells was monitored in time using flow cytometry. On day 0, T cells (or CAR-T cells directly after transduction) were cultured in the presence of 100 U/ml of IL-2 alone. After 24 h, the human T-activator CD3/CD28 beads were added, and cells were cultured for the next 6 days. To monitor PD-L1 surface expression in NK-92 cells upon antigen stimulation, parental, CD19-CAR NK-92, and PD-L1-CAR NK-92 cells were seeded onto a 12-well plate at density 1×10^6 mln per well in 1 ml full RPMI-1640 medium. Raji PD-L1 cells were added to the effector cells in a 1:1 effector:target ratio in a 2 ml full RPMI-1640 medium. After 24 h and 48 h co-culture, the cells were washed with PBS and stained with viability dye (Zombie NIR, cat. No., BioLegend, dilution 1:400), followed by CD56 (cat. No. 318328, Biolegend, clone HCD56, dilution 1:50) and PD-L1 staining (cat. No. 12-5983-42, eBioscience, clone MIH-1, dilution 1:100). The PD-L1 expression was analyzed by flow cytometry.

PD-L1 and HER-2 expression on target and effector cells

PD-L1 expression on target or effector cells was assessed using anti-PD-L1 antibody either clone MIH-1 antibody (Thermo Fisher Scientific Cat# 12-5983-42, RRID:AB_11042286, dilution 1:100) or clone 29E.2A3 (BioLegend Cat# 329734, RRID:AB_2629580, dilution 1:100). HER-2 expression on target cells was assessed using anti-HER2 antibody (BioLegend Cat# 324408, RRID:AB_2262301, dilution 1:100).

Functional assays (degranulation and cytokine release)

Degranulation of cytotoxic T cells and NK cells was performed by evaluating the expression of CD107a (LAMP-1) on the surface of T/NK cells with anti-CD107a antibody conjugated with fluorochrome. To this end, target cells were seeded at the round bottom 96-well plate in the presence of effector cells and CD107a-PE antibody (BD Biosciences Cat# 555801, RRID:AB_396135, dilution 1:40) and GolgiStop (BD Biosciences Cat# 554724, RRID:AB_2869012, dilution 1:250) and/or GolgiPlug (BD Biosciences Cat# 555029, RRID:AB_2869014, dilution 1:200). Typically, a 2:1 effector to target ratio was used for the functional assay. After 4 hours, cells were spun down, washed, and stained for the extracellular markers – CD3 for T cells (BD Biosciences Cat# 562426, RRID:AB_11152082, dilution 1:100), CD56 for NK, NK-92 cells (Miltenyi Biotec Cat# 130-113-308, RRID:AB_2726086, dilution 1:50) and Fixable Viability Dye to exclude dead cells (cat. No. L34963, Thermo Scientific, dilution 1:100). After fixation and permeabilization, intracellular staining for cytokines using anti-IFNγ (BD Biosciences Cat# 341117, RRID:AB_2264629, dilution 1:100) and anti-TNFα (BD Biosciences Cat# 557647, RRID:AB_396764, dilution 1:100) antibodies conjugated with fluorochromes was performed. Degranulation and cytokine production by effector cells was assessed using flow cytometry.

Flow cytometry-based killing assay

For fratricide cytotoxicity assay, target T cells were pre-stimulated for 24 h with human T-activator CD3/CD28 beads to induce PD-L1 expression on their surface. To separate target cells from effectors, target cells were stained with Cell Trace CFSE and effectors were stained with Cell Trace Violet (CTV). T cells were mixed and incubated with unmodified T cells, CD19-CAR or PD-L1 CAR T-cells for 24 h at E:T ratio 2:1. Propidium iodide (PI) was used to discriminate live/dead cells. Cytotoxicity of effector cells was evaluated as an increase in a percentage of green-CFSE (or violet-CTV) positive, PI positive target cell population

Preparation of T cells/NK-92 parental cells or PD-L1-CAR-T/NK-92 cell supernatants

MDA-MB-231 parental or sgPD-L1 breast cancer cells were seeded onto a 6-well plate at a cell density 1×10^6 mln per well in 2 ml full RPMI-1640 medium and left overnight to attach. Next, the medium was removed and replaced with either unmodified or PD-L1-CAR T or NK-92 parental or PD-L1-CAR NK-92 cells. The effector cells were added at a 1:1 E:T ratio and co-incubated for the next 24 h. Afterward,

the supernatants were collected, centrifuged at 2 000 × *g* for 5 min, and left for further experiments at -20°C.

Induction of PD-L1 on the cell surface of target cells

Cell lines were seeded onto the 24-well (or 12-well) plate at a cell density 1-2 × 10^5 per well. After 24 h, the medium was removed and changed either for the control (a fresh portion of RPMI-1640 full medium), or 25 ng/ml IFNγ, or conditioned supernatants from the 24-hour co-incubation cultures of unmodified T cells or PD-L1-CAR T cells with the target parental or sgPD-L1 MDA-MB-231 cells and incubated for the next 48 hours. Moreover, MCF-7 and SKBR-3 cells were co-incubated with HER-2-CAR T cells. For sequential killing experiments, MCF-7 cells were preincubated with or without 100 µg/ml trastuzumab for 24 h, and then incubated alone or together with T cells or HER-2 CAR T cells at an E:T ratio of 0.5:1. Then, the cells were detached with trypsin, washed with PBS, and stained with viability dye (Zombie NIR, cat. No. 423105, BioLegend, dilution 1:400 or Fixable Viability Stain 510, cat. No. 564406, BD Bioscience, dilution 1:20000) followed by PD-L1 staining (cat. No. 12-5983-42, eBioscience, clone MIH-1, dilution 1:100). The PD-L1 expression was analyzed by flow cytometry.

RTCA-based killing assay

For RTCA assay, adherent target cells were seeded onto 16-well E-Plates (ACEA Biosciences) at a cell density 3 × 10^4 per well in 100 µl of the appropriate medium and monitored in the incubator at 37°C (5% CO_2, 95% humidity) for 24 h with the xCELLigence impedance-based RTCA system (Acea Biosciences). Next, the medium was removed and replaced with RPMI-1640 full medium containing control or CAR-modified effector cells (primary T cells or NK92 cells) at various E:T ratios. For some experiments, 0.4 or 0.8 mg/ml atezolizumab (ATEZO, Roche) was added. The cells in the E-Plates were monitored with the RTCA system for the next 12-20 h. For the sequential killing, the first portion of CAR-T cells was added, and after 6-hour of impedance measurement, it was supplemented with the another portion of appropriate CAR-T cells and left for the next 16 h. Sequential killing experiments were performed in the presence or absence of 100 µg/ml of trastuzumab. The analysis of the results was performed using RTCA Software Pro (ACEA Biosciences). The impedance changes were normalized to the moment of the effector cells addition and plotted over time as normalized cell index.

Animals studies

The in vivo experiments were performed in the Animal Facility of the Medical University of Warsaw in accordance with the EU Directive 2010/63/EU and the Polish legislation for animal experiments of the Polish Ministry of Science and Higher Education (February 26, 2015) and approved by the Second Local Ethics Committee for the Animal Experimentation, Warsaw University of Life Sciences in Warsaw (for MDA-MB-231 study - WAW2/111/2019 and for MCF-7 - WAW2/108/2021). For the study, 6-12 old

female NSG (NOD.Cg-Prkdcscid Il2rgtm1Wjl/SzJ) mice (The Jackson Laboratory), were used. The experiments were carried out in SPF animal facility with IVC systems. To avoid confounders, all mice were labeled and kept in tagged cages. The cages had an assigned, unchanging place in the rack. Results obtained from individual mice or mesurement means according to the treatment method are presented. The blinding was not applied, however, the experiments were supervised by two experimenters, independently. The distribution of mice to the experimental groups was random and no animals were excluded during the experiment. The sample size was determined based on the assumed increase in tumor diameter. The experimental group size was calculated by power analysis or resource equation approach as described in [7].

In vivo treatments

MDA-MB-231 cells (1×10^6) were inoculated into the second, left mammary fat pad of NSG mice in 50% Matrigel (BD, USA), on day 0 of the experiment. Subsequently, on days 8 and 15, 5×10^6 PD-L1-CAR-T, CD19-CAR-T, or unmodified T cells were administrated intratumorally. Control mice were injected with PBS. Tumor growth was monitored two times per week with calipers and the tumor volume was calculated using the formula: volume = (length × width2)/2. The mice were sacrificed when the tumors reached 12 mm in diameter. Total number of mice used within this study – 38.

In the case of the experiments involving MCF-7 cells the slow-release pellets containing 17β-estradiol (Innovative Research of America, USA), were implanted subcutaneously four days before tumor cells inoculation. Then NSG mice were injected with 3×10^6 MCF-7 pLVX or MCF-7 PD-L1 cells into the second, left mammary fat pads in 50% Matrigel (BD, USA). Finally, the 5×10^6 PD-L1-CAR-T or control T cells or PBS were injected intratumorally on 24, 27, and 30 day of the experiment. Tumors were measured with calipers once per week. Total number of mice used within this study – 35.

Luciferase-based killing assays

HEK-293T cell lines (HEK-293T pLVX (PD-L1 $^{low/null}$) and HEK-293T PD-L1 (PD-L1^{positive})), previously modified to express luciferase reporter gene (Red-Luc), were seeded onto the 96-well black plates with a clear bottom (Perkin Elmer) at a cell density 2.5×10^4 per well in 100 µl of full DMEM medium in triplicates and allowed to adhere for 24 h. The next day, 100 µl of the medium was removed and replaced with 100 µl of culture medium with suspended effector cells. The cells were co-incubated at various effector to target ratios for 18 h at 37°C (5% CO_2, 95% humidity). Bright-Glo™ Luciferase Assay System (cat. No. E2610, Promega) was used for bioluminescence readout. 100 µl of the reagent was added to each well with the co-cultured cells. The plate was incubated for 5 min in darkness at 25°C, and then the luminescence was measured using Victor Plate Reader (Perkin Elmer).

Live imaging experiments (BM-MSC killing)

BM-MSCs were seeded onto the 96-well black plates with clear bottom (PerkinElmer) at a cell density 5×10^3 per well in 100 µl of DMEM medium supplemented with 10% FBS and 1% Pen/Strep in duplicates and allowed to adhere for 24 h. For the next 24 h, the cells were cultured in the presence or absence of 25 ng/ml of IFNγ. Afterward, to reduce background fluorescence, the medium was replaced with FluoroBrite DMEM (Life Technologies) medium supplemented with 10% FBS, 1% Pen/Strep, and 2 mM L-glutamine, and the effector NK-92 parental or PD-L1-CAR-modified cells were added at the E:T ratio of 0.5:1 for 8 h. To assess apoptosis progression, the 0.5 µM of CellEvent Caspase-3/7 Green Detection Reagent (cat. No. C10427, Thermo Scientific) was added. The apoptosis of the BM-MSCs in the presence of NK-92 cells was monitored in real-time using Cytation™ 1 Cell Imaging Multi-Mode Reader with 15 min intervals. Consecutive images were acquired at defined time points for 8 h using 488-nm excitation and collecting fluorescence emission using a 530/30 bandpass filter. The number of Caspase-3/7 positive cells was calculated using Gene 5 3.04 software (BioTek, Agilent).

Cytokine array

The cytokine arrays were co-incubated with the supernatants collected from the 24-h co-cultures of MDA-MB-231 parental breast cancer cells with T cells/NK-92 parental (control) or PD-L1-CAR T/NK-92 cells overnight at 4°C with shacking. The array procedure was performed using R&D Systems Human Cytokine Array, Panel A (cat. No. ARY005, R&D Systems) according to the manufacturer's protocol. The signals on the developed membrane were visualized by chemiluminescence detection using ChemiDoc Imaging System (Bio-Rad Laboratories, Hercules, CA, USA). The densitometric analysis was performed using Image Lab software (Bio-Rad Laboratories, RRID:SCR_014210, Hercules, CA, USA) and GraphPad Prism 8 (GraphPad Software, RRID:SCR_002798, San Diego, CA, USA).

Statistical analysis

Statistical analysis was performed with GraphPad Prism 8 (GraphPad Prism, RRID:SCR_002798). Results are shown as the mean value ± standard deviation (SD) unless otherwise stated. Normality was checked using the Shapiro-Wilk test. The comparison between two groups for paired samples was analyzed using Student t-test or Wilcoxon test and for unpaired samples using unpaired t-test or Mann-Whitney test depending on data distribution. For the in vivo experiment, a two-way ANOVA test was applied. The P values of less than 0.05 were considered significant.

Supplementary Figure Legends

Suppl. Figure 1. Expression of PD-L1 in cancer cell lines.

A. *The representative Western Blot analysis of PD-L1 expression (PD-L1 antibody, clone E1L3N, cat. No. 13684, Cell Signaling, dilution 1:1000) in ovarian (A2774, A2780, OvaCa3, MDAH), cervical (HeLa), Hodgkin lymphoma (L540, L428, SUP-HD1, HDLM-2), non-small cell lung cancer (A549) and melanoma (M407, M257) cancer cell lines. The experiment was repeated three times.*

B. *Representative density plots and histogram overlays illustrating PD-L1 expression (red) against a background from isotype control (grey) for the HCC1806 breast cancer cell line using flow cytometry. The staining was performed using an anti-PD-L1 antibody (cat. no. 12-5983-42, eBioscience, clone MIH1, dilution 1:100). Numbers on the density plots indicate the percentage (%) of PD-L1 positive cells. The experiment was repeated at least two times.*

C. *Representative density plots and histogram overlays illustrating PD-L1 expression (red) against a background from isotype control (grey) for MCF-7 (upper panel) and MDA-MB-231 (lower panel) breast cancer cell lines using flow cytometry. The staining was performed using an anti-PD-L1 antibody (cat. No. 329734, BioLegend, clone 29E.2A3, dilution 1:100). Numbers on the density plots indicate the percentage (%) of PD-L1 positive cells. The experiment was repeated at least three times.*

Suppl. Figure 2. Time-dependent enrichment in PD-L1-CAR-T presence in the population of the transduced T cells, transient NK cells modifications using mRNA electroporation technique and selection of PD-L1-CAR NK-92 positive population by FASC sorting.

A. *Flow cytometry analysis of the surface PD-L1-CAR expression was assessed 3 and 10 days following the lentiviral transduction with PD-L1-CAR encoding vector. Unmodified and PD-L1-CAR T cells were washed with EasySep buffer (PBS supplemented with 2% FBS and 1 mM EDTA) and stained using goat anti-human IgG, Fcγ fragment specific antibody conjugated with Alexa Fluor 647 (cat. No. 109-606-098, Jackson ImmunoResearch) diluted 1:200 in EasySep buffer.*

B. *Flow cytometry analysis of the surface PD-L1-CAR expression in NK cells after mRNA electroporation form two experiments with three and two individual donors (n = 5, bar graph, left side) and representative flow cytometry density plots of PD-L1-CAR expression on the surface of mock (unmodified) and PD-L1-CAR-NK cells detected by anti-human IgG, Fcγ fragment specific antibody (right side). Numbers on the density plots indicate the percentage (%) of PD-L1-CAR positive cells.*

C. *PD-L1-CAR positive NK-92 cells were stained with anti-human IgG antibody recognizing Fcγ fragment and sorted based on their PD-L1-CAR expression using fluorescence-activated cell sorting (FACS) flow cytometry. Numbers on the density plots indicate the percentage (%) of PD-L1-CAR positive cells.*

Suppl. Figure 3. The expression pattern of PD-L1 after CAR T cells stimulation

A. *The scheme depicting the modular structure of CD19 CARs used within this study (for details see Materials and Methods Section).*

B. *PD-L1 expression on PD-L1- and CD19-CAR T cells stimulated by human T-activator CD3/CD28 beads and IL-2 (100 U/ml). CAR T cells were cultivated in the presence of 100 U/ml of IL-2 alone (Day 0)*

or together with human T-activator CD3/CD28 beads (Day 1-6). Day 1 represents the first day after the stimulation of T cells with human T-activator CD3/CD28 beads. PD-L1 staining was performed on consecutive days using an anti-PD-L1 (clone MIH1, dilution 1:100). The experiment was repeated in duplicate two times.

C. *Flow cytometry analysis of fratricide killing performed by PD-L1-CAR T cells. The left-hand panel depicts the scheme and timeline of the experiment. The right-hand panel presents the killing potential of effectors (CTV stained PD-L1-CAR-T or CD19-CAR T cells) against unmodified T cells derived from the same donor and stimulated with human T-activator CD3/CD28 beads and IL-2 (100 U/ml) for 24 h and stained with CFSE prior to the experiment. Effector cells (PD-L1-CAR-T cells or CD19-CAR-T cells) were added to the targets - unmodified T cells at the E:T ratio of 2:1 in the absence or presence of 0.6 mg/ml atezolizumab. After 24 h propidium iodide was added and the percentage (%) of the CFSE and PI positive cells was assessed with flow cytometry. The experiment was repeated in duplicates two times.*

D. *The representative Western Blot analysis of PD-L1 expression (PD-L1 antibody, clone E1L3N, cat. No. 13684, Cell Signaling, dilution 1:1000) in PD-L1- and CD19-CAR T cells stimulated by human T-activator CD3/CD28 beads and IL-2 (100 U/ml). The lysates were collected collected in day 0 as well as in days 1, 4, and 7 post stimulation and day 8 after beads re-stimulation. The experiment was repeated two times. Bands were quantified by densitometry; the signal for the PD-L1 band was normalized to the corresponding actin band (lower panel).*

E. *qPCR analysis of PD-L1 mRNA expression in unmodified T cells, PD-L1- and CD19-CAR T cells stimulated by human T-activator CD3/CD28 beads and IL-2 (100 U/ml). mRNA was collected on day 0 as well as in days 1, 4, and 7 post-stimulation and day 8 after beads re-stimulation. The experiment was repeated in duplicate two times.*

F. *Phenotypic characteristics of PD-L1-CAR T cells. The differentiation and exhaustion markers on the surface of unmodified T cells, CD19-CAR T, and PD-L1-CAR T cells were performed on days 4 and 10 after CARs transduction and analyzed by flow cytometry.*

Suppl. Figure 4. The efficacy of PD-L1-CAR NK-92 and primary NK cells against breast cancer cells and generation of the PD-L1-knockout derivative of MDA-MB-231 cell line and the PD-L1-overexpressing derivative of MCF-7 cell line and their susceptibility to PD-L1-CAR NK-92 cells cytotoxic activity.

A. *Functional and cytokine release assays of PD-L1-CAR NK-92 cells targeted against MCF-7 (PD-L1$^{low/-}$) or MDA-MB-231 (PD-L1^{+}) cancer cell lines were assessed by flow cytometry. Degranulation assay, assessed by CD107a staining (left panel), and IFNγ release (right panel) were measured after 5 h of co-incubation of target and effector cells at the E:T ratio of 2:1. The experiment was repeated in duplicates. Bars represent the ± SD with the p-values derived from unpaired t-test (*p < 0.05, **p < 0.01).*

B. *Degranulation (measured by CD107a, left panel) and IFNγ release (right panel) assays of PD-L1-CAR NK cells targeted against MDA-MB-231 (PD-L1^{+}) cancer cell line were assessed by flow cytometry. The experiment was performed two times, with three and two individual donors (n = 5). Bars represent the mean value ± SD. The differences between groups were analyzed using unpaired t-test (*p < 0.05, ***p < 0.001).*

C. *Representative flow cytometry density plots showing efficient ablation of PD-L1 by CRISPR/Cas9 in MDA-MB-231 cells. PD-L1 surface presence was assessed using anti-PD-L1 antibody (clone 29E.2A3).*

D. *Western blot analysis of PD-L1 expression in unmodified (parental) and two knockouts (KO clone 1 and 2) MDA-MB-231 cells. β-actin was used as a loading control. The experiment was repeated three times.*

E. *Representative flow cytometry histograms showing overexpression of PD-L1 by lentiviral transduction of MCF-7 cells (MCF-7 PD-L1) compared to empty vector-modified MCF-7 cells (MCF-7 pLVX). PD-L1 surface presence was assessed using anti-PD-L1 antibody (clone 29E.2A3).*

F. *Western blot analysis of PD-L1 expression in control (parental, pLVX) and PD-L1 overexpressing MCF-7 cells. β-actin was used as a loading control. The experiment was repeated three times.*

G. *Degranulation and IFNγ release assays of PD-L1-CAR NK-92 cells targeted against MCF-7 pLVX (PD-L1$^{low/-}$) and MCF-7 PD-L1 or MDA-MB-231 sgNTC (PD-L1^{+}) or MDA-MB-231 sgPD-L1 (PD-L1^{-}) cancer cell lines were assessed by flow cytometry. Degranulation assay assessed by CD107a (left panel) staining and IFNγ release (right panel) were measured after 2.5 h of co-incubation of target and effector cells at the E:T ratio of 2:1. The experiment was repeated in duplicates. Bars represent the mean value ± SD. The differences between control and CAR-affected groups were analyzed using unpaired t-test (*$p < 0.05$, **$p < 0.01$, ***$p < 0.001$).*

H. *The cytotoxic activity of PD-L1-CAR-T cells against T47D cell line, at the E:T ratio of 1:1 and 2:1 were measured by impedance analysis. Samples were internally normalized for the cell index value measured before CAR-T cells addition (Normalized Cell Index plots). Representative average impedance curves from 2 wells are shown. The experiment was repeated in duplicates two times.*

I. *The growth of MDA-MB-231 tumors injected with PBS (control) vs tumors injected with T cells, CD19- or PD-L1-CAR T cells in mice (initial n=8 mice/group). A single tumor volume plots vs. the number of days post first CAR injection are depicted.*

J. *The growth of MCF7-pLVX tumors injected with PBS (control) vs. tumors injected with T cells or PD-L1-CAR T cells in mice (initial n=6-7 mice/group), left panel. The growth of MCF7-PD-L1 tumors injected with PBS (control) vs. tumors injected with T cells or PD-L1-CAR T cells in mice (initial n=4-7 mice/group), right panel.*

Suppl. Figure 5. Induction of PD-L1 expression on the target cells after different stimuli.

A. *PD-L1 expression induced on cancer cells by supernatant from activated CAR T cells was assessed by flow cytometry. The control (only medium) and conditioned supernatants from the 24-hour co-cultures of control (unmodified) or PD-L1-CAR T cells with the target MDA-MB-231 cells were transferred onto the culture of sgNTC and sgPD-L1 MDA-MB-231 and incubated for 24 hours. Next, PD-L1 surface presence was assessed by flow cytometry using anti-PD-L1 antibody (clone MIH1). The experiment was repeated two times.*

B. *IFNγ induced expression of PD-L1 on breast cancer cells (MDA-MB-231 and MCF-7) was assessed by flow cytometry. PD-L1 staining was performed using anti-PD-L1 antibody (clone MIH1).*

C. *Representative images of different subpopulations of macrophages (M0, M0+IFNγ, M2) stained for PD-L1 assessed by immunocytochemistry assay using Cytation 1 Cell Imaging Multi-Mode Reader (Biotek, Agilent). The control (only medium) and conditioned supernatants from the 24-hour cultures of MDA-MB-231, MDA-MB-231 sgPD-L1, or MDA-MB-231 cells co-incubated with CAR PD-L1 T cells were transferred onto different subpopulations of macrophages and incubated for 48 hours. Next, PD-L1 surface presence was assessed using the anti-PD-L1 antibody (clone MIH1). The signal was developed using AF488-conjugated secondary antibody (green), and nuclei were counterstained with DAPI (blue), scale bar: 100 µm. The background fluorescence was removed*

and the low threshold for green fluorescence was set to create a mask of the area covered by macrophages. Bar graphs represent the mean fluorescent intensity of PD-L1 within the thresholded area. The experiment was performed in duplicates with two donors (n = 2).

D. *The representative Western blot results of cytokine array assay performed with the supernatants collected from control unmodified T or PD-L1-CAR T (left hand panels) and NK-92 or PD-L1-CAR NK-92 cells (right-hand panels) co-cultured with MDA-MB-231 breast cancer cells for 24 h at 1:1 E:T.*

Suppl. Figure 6. The efficacy of HER-2-CAR T cells against MCF-7 breast cancer cells.

A. *The scheme depicting the modular structure of HER-2-CAR.*

B. *Flow cytometry analysis of HER-2-CAR expression in control (upper panels) and lentiviral transduced T cells (lower panels). HER-2-CAR expression was detected using anti-human CD34 antibody (cat. No. 17-0349-42, eBioscience™). Numbers on the density plots indicate the percentage (%) of HER-2-CAR positive cells. The experiments were repeated at least three times.*

C. *The potential of killing tumor cells by HER-2-CAR-T cells was measured by impedance analysis for MCF-7 cells. Cells were left to adhere and to form a monolayer on the E-plates for 24 h. The next day, HER-2-CAR-T cells were added to the monolayers for 12 hours at the indicated E:T ratios. Representative impedance curves were shown. The experiment was repeated in duplicate two times.*

D. *HER-2 surface expression on MCF-7 and SKBR-3 (as positive control) cells was assessed by flow cytometry with an anti-HER-2 antibody (Cat. No. 324408, BioLegend). Bar graph showing the average mean fluorescent intensity of PD-L1 expression in MCF-7 and SKBR-3 cells compared to the isotype control. Bar graph represents the average MFI from two experiments performed in duplicates.*

Suppl. Figure 7. The killing potential of CAR-PD-L1-bearing NK-92 cells towards the bone marrow-derived mesenchymal stem cells and generation of the PD-L1 overexpressing HEK-293T cell line.

A. *BM-MSC cells were cultured in the presence or absence of 25 ng/ml of IFNγ for 24 h. Next, the effector NK-92 parental or CAR PD-L1 cells were added at the E:T ratio of 0.5:1 for 8 hrs. To assess apoptosis progression, the CellEvent Caspase-3/7 Green Detection Reagent was added. Apoptosis of the BM-MSC cells was monitored in real-time using Cytation™ 1 Cell Imaging Multi-Mode Reader in 15 min intervals. The number of Caspase-3/7 positive cells was calculated using Gene 5 3.04 software (BioTek, Agilent). The experiment was repeated in duplicate for six individual BM-MSC donors. Statistical analysis was performed within a time-point. Normality was checked using the Shapiro-Wilk test. The p-values derived from the Student t-test are presented for BM-MSC + IFNγ + CAR-PD-L1 NK-92 (dark red line) comparing to BM-MSC + CAR-PD-L1 NK-92 (red line) samples (*p < 0.05; **p < 0.01). Representative images were showing the detection of apoptosis of BM-MSC (preincubated with or without 10 ng/ml IFNγ) upon incubation with CAR PD-L1-bearing NK-92 cells using CellEvent Caspase-3/7 Green Detection Reagent in selected time points (0, 2, 4, 6, 8 h).*

B. *The generation of the PD-L1 overexpressing derivative of HEK-293T Red-Luc cell line (containing stably transduced Red-Luc luciferase) by lentiviral transduction. Representative flow cytometry histograms showing surface overexpression of PD-L1 on HEK-293T Red-Luc (HEK-293T-PD-L1) compared to control (HEK-293T-pLVX, transduced with empty vector). The PD-L1 surface expression was assessed using anti-PD-L1 antibody (clone MIH1).*

1 Demaison C, Parsley K, Brouns G, Scherr M, Battmer K, Kinnon C *et al*. High-level transduction and gene expression in hematopoietic repopulating cells using a human immunodeficiency [correction of imunodeficiency] virus type 1-based lentiviral vector containing an internal spleen focus forming virus promoter. *Hum Gene Ther* 2002; 13: 803-813.

2 Priceman SJ, Tilakawardane D, Jeang B, Aguilar B, Murad JP, Park AK *et al*. Regional Delivery of Chimeric Antigen Receptor-Engineered T Cells Effectively Targets HER2(+) Breast Cancer Metastasis to the Brain. *Clin Cancer Res* 2018; 24: 95-105.

3 Köksal H, Baken E, Warren DJ, Løset GÅ, Inderberg EM, Wälchli S. Chimeric antigen receptor preparation from hybridoma to T-cell expression. *Antibody Therapeutics* 2019; 2: 56-63.

4 Soneoka Y, Cannon PM, Ramsdale EE, Griffiths JC, Romano G, Kingsman SM *et al*. A transient three-plasmid expression system for the production of high titer retroviral vectors. *Nucleic Acids Res* 1995; 23: 628-633.

5 Bajor M, Zych AO, Graczyk-Jarzynka A, Muchowicz A, Firczuk M, Trzeciak L *et al*. Targeting peroxiredoxin 1 impairs growth of breast cancer cells and potently sensitises these cells to prooxidant agents. *Br J Cancer* 2018; 119: 873-884.

6 Sanjana NE, Shalem O, Zhang F. Improved vectors and genome-wide libraries for CRISPR screening. *Nat Methods* 2014; 11: 783-784.

7 Charan J and Kantharia ND. How to calculate sample size in animal studies? J Pharmacol Pharmacother. 2013 Oct;4(4):303-6
