# Supplementary figures and images for: PD-L1 CAR effector cells induce self-amplifying cytotoxic effects against target cells
Source: J Immunother Cancer. 2022 Jan 24;10(1):e002500. doi: 10.1136/jitc-2021-002500 (PMC8796262; doi:10.1136/jitc-2021-002500)

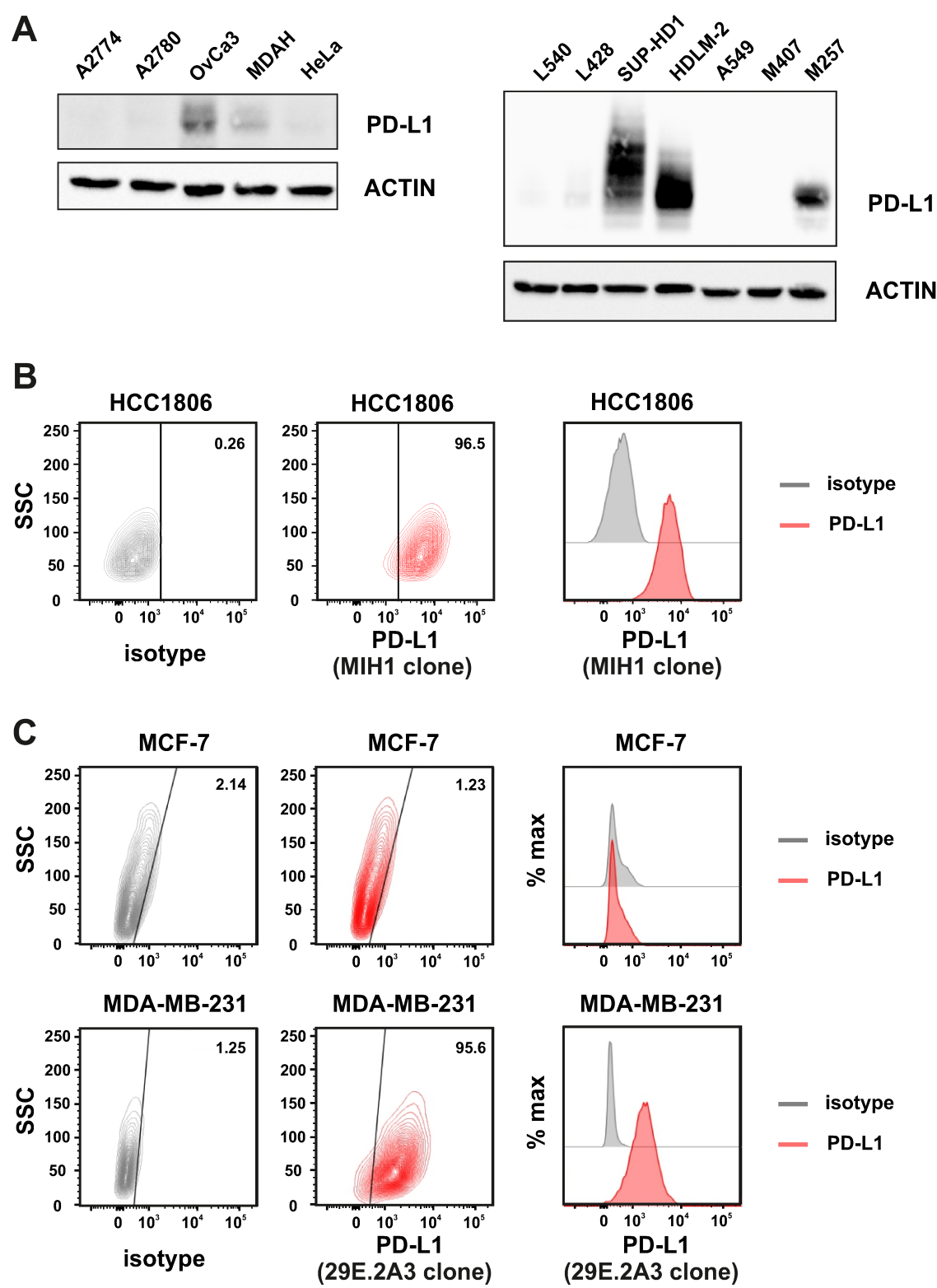

Suppl Fig.1

A

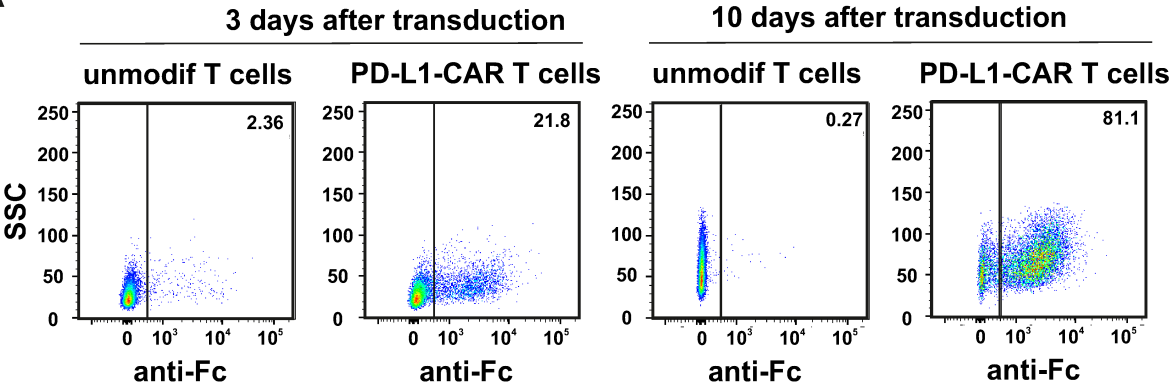

B

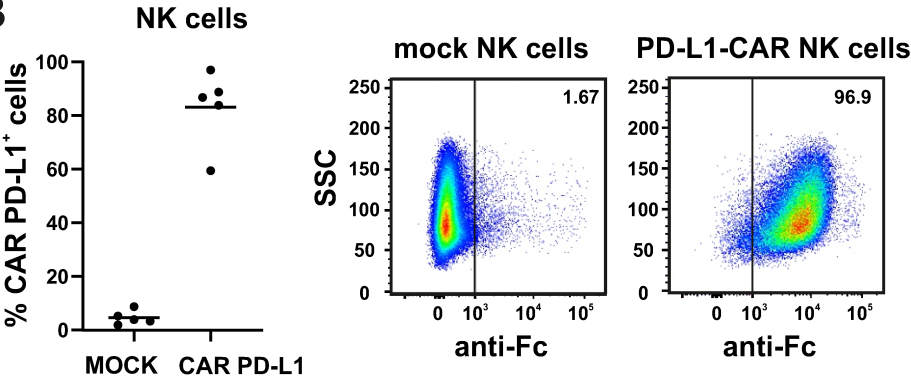

C

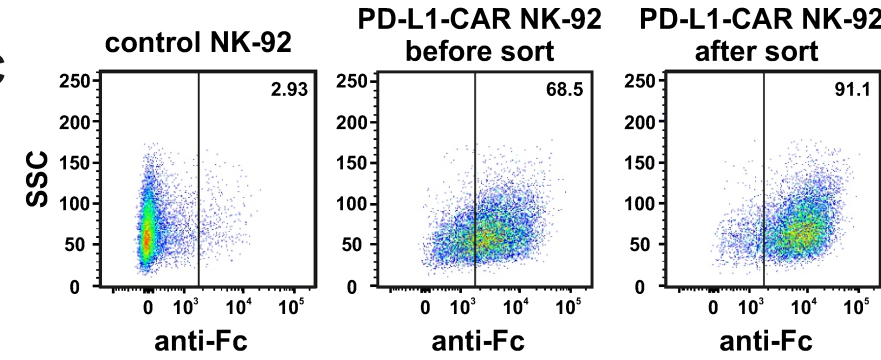

Suppl Fig.2

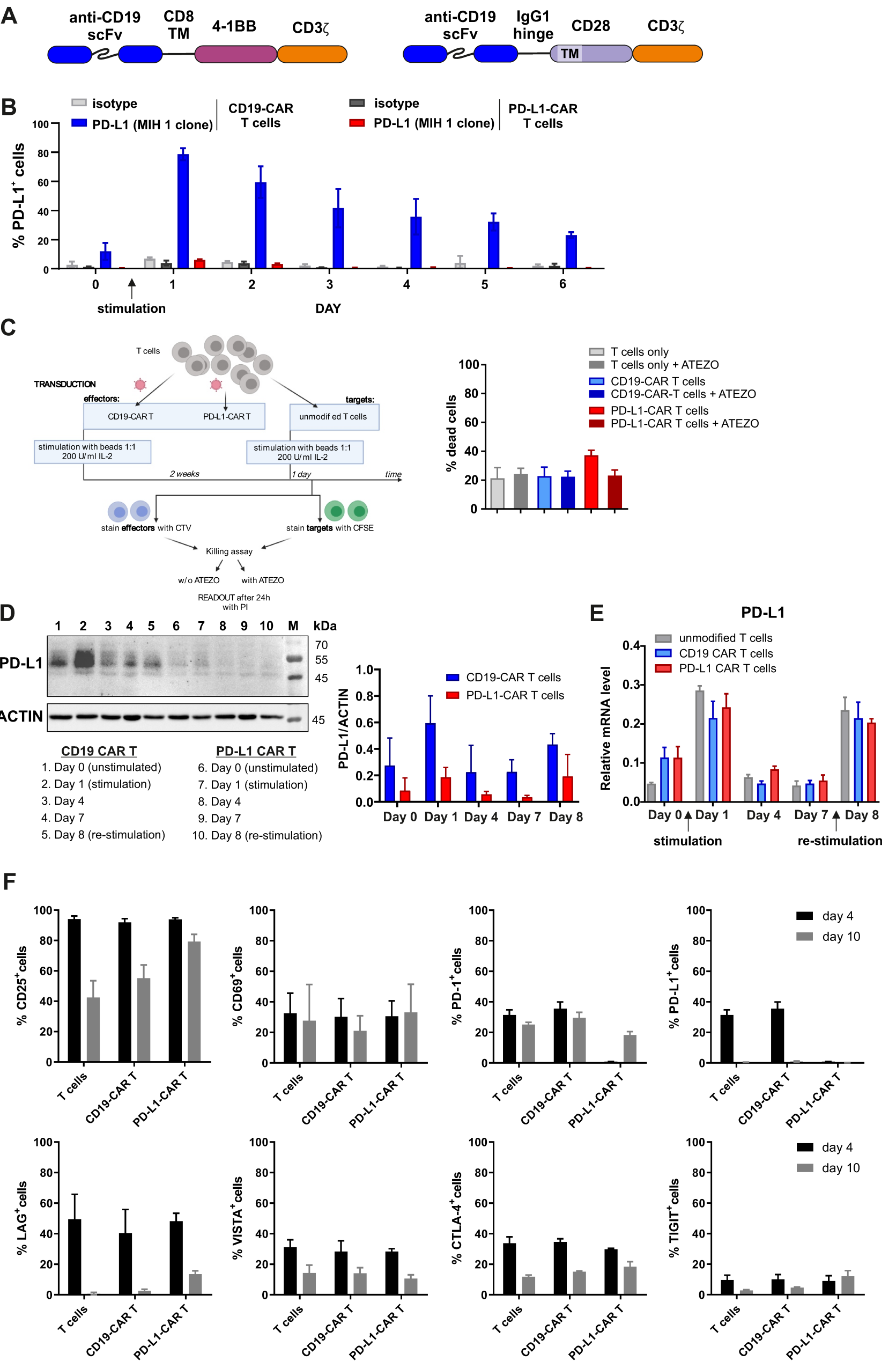

Suppl Fig.3

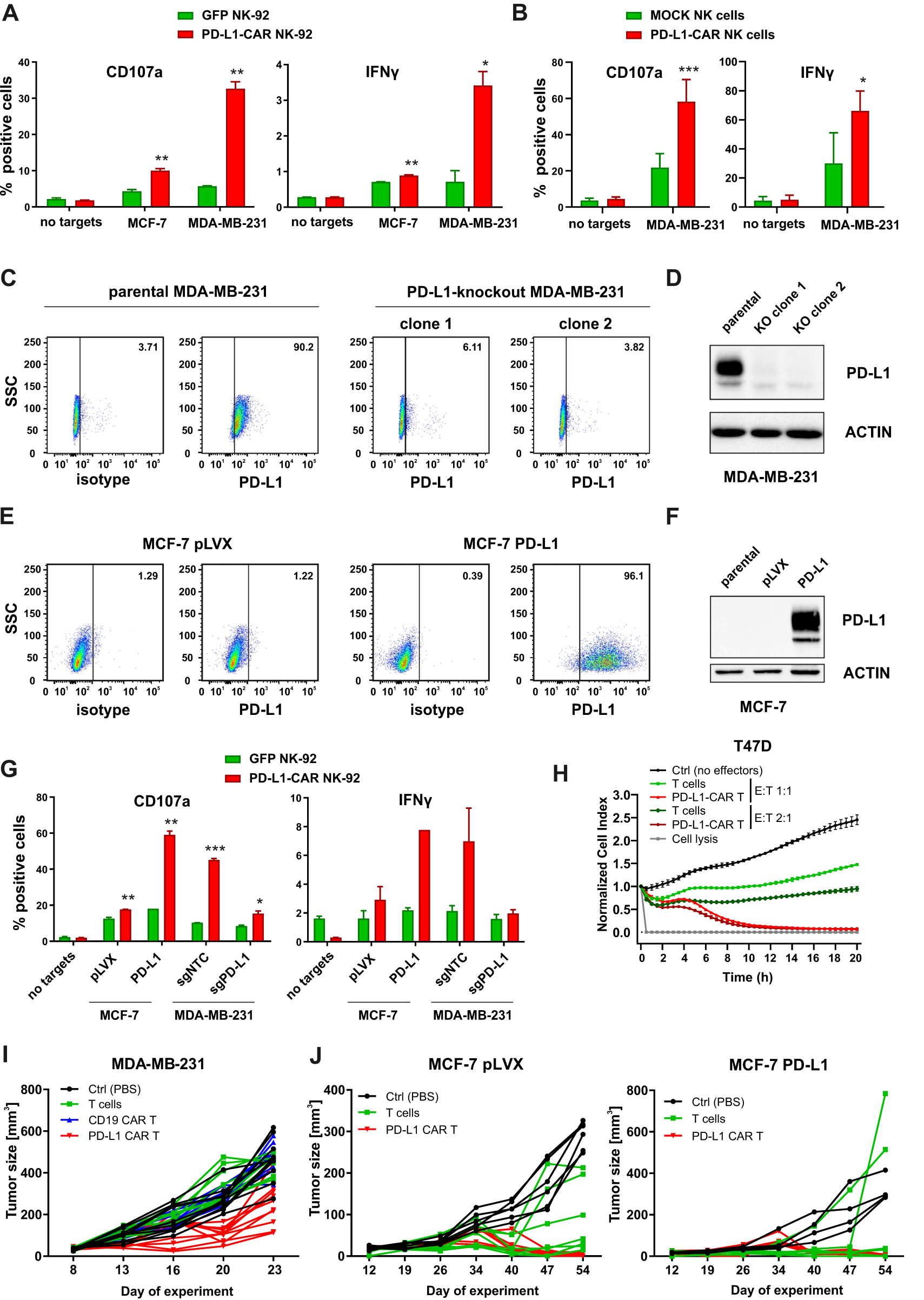

Suppl Fig.4

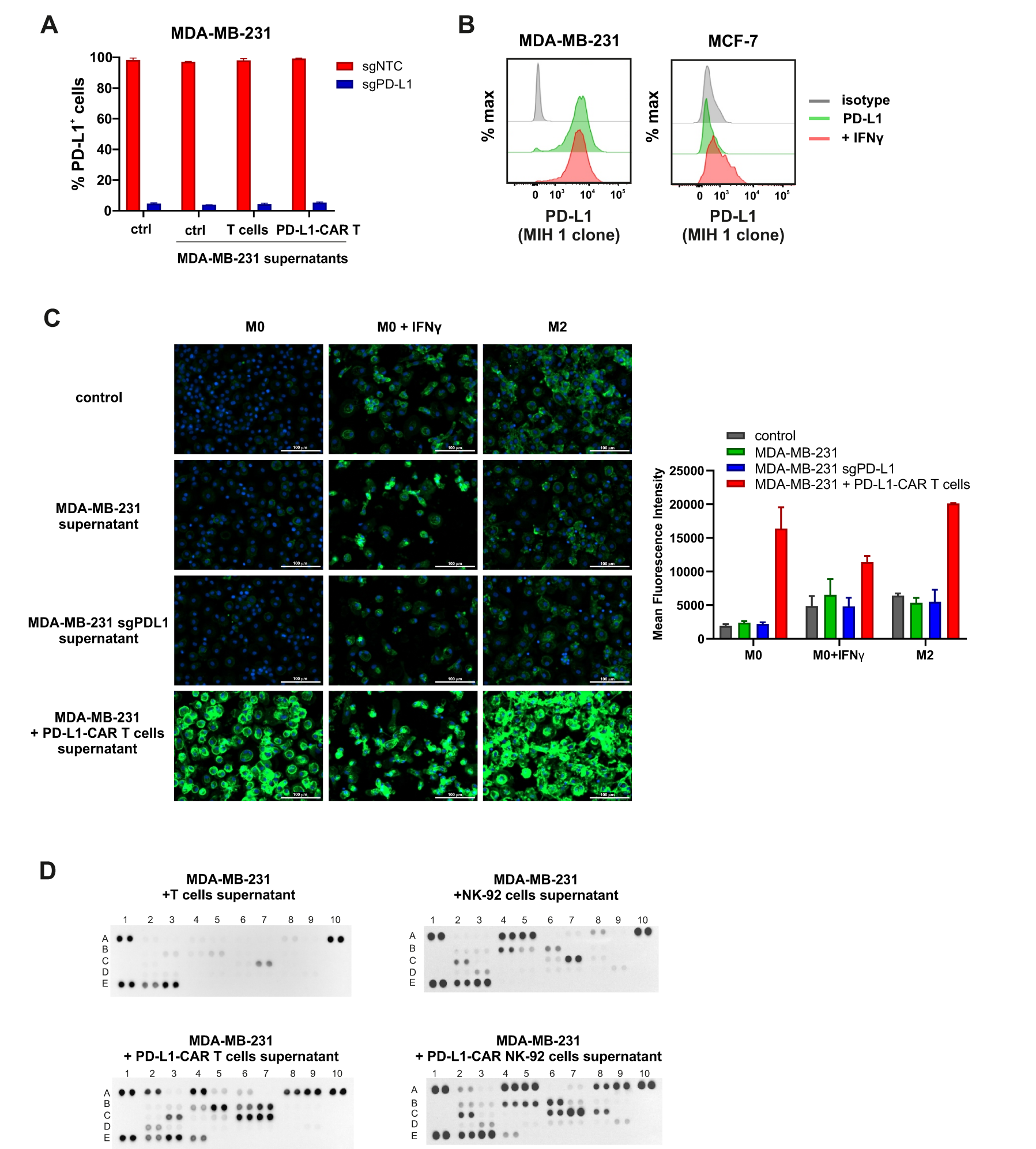

Suppl Fig.5

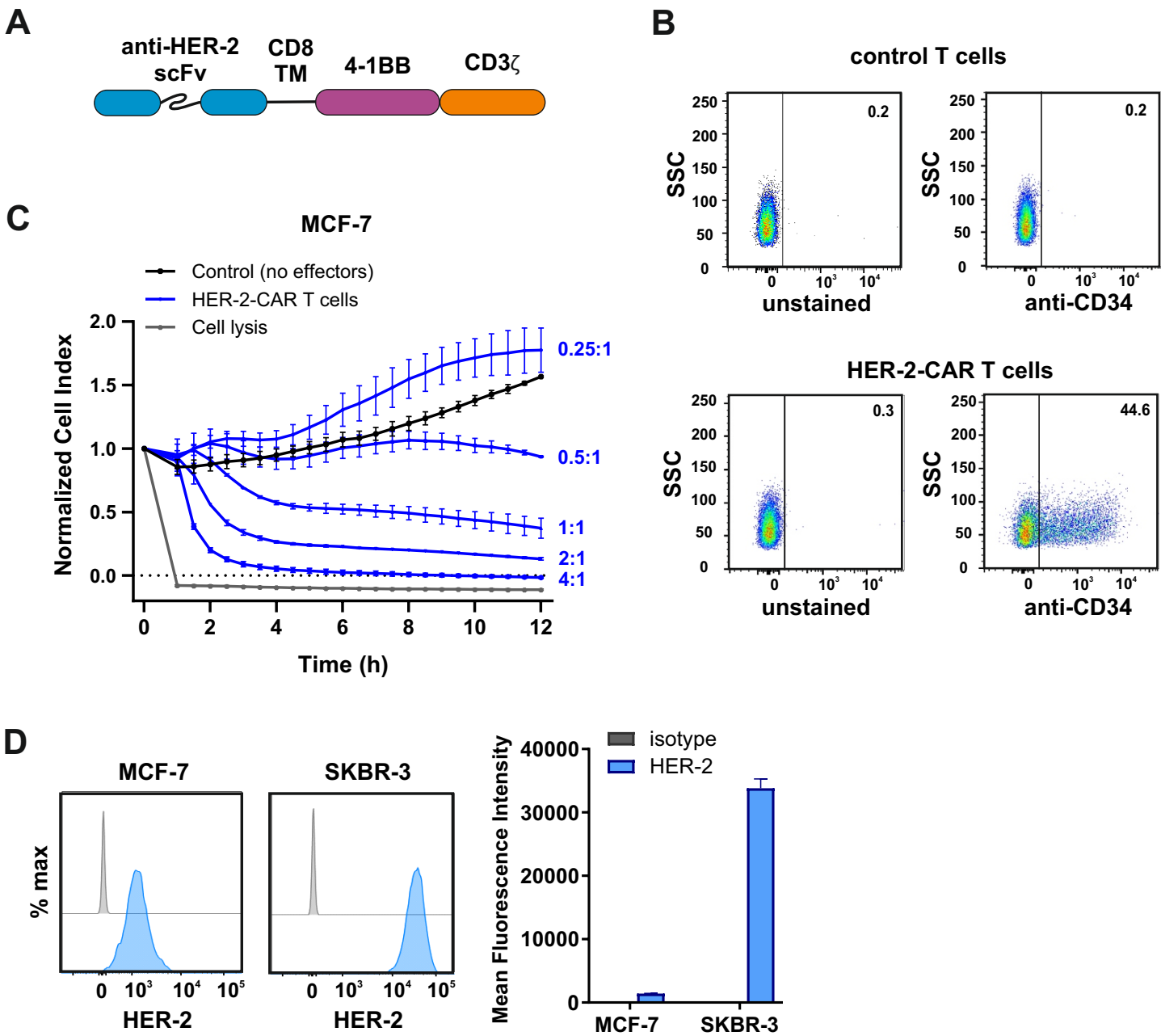

Suppl Fig.6

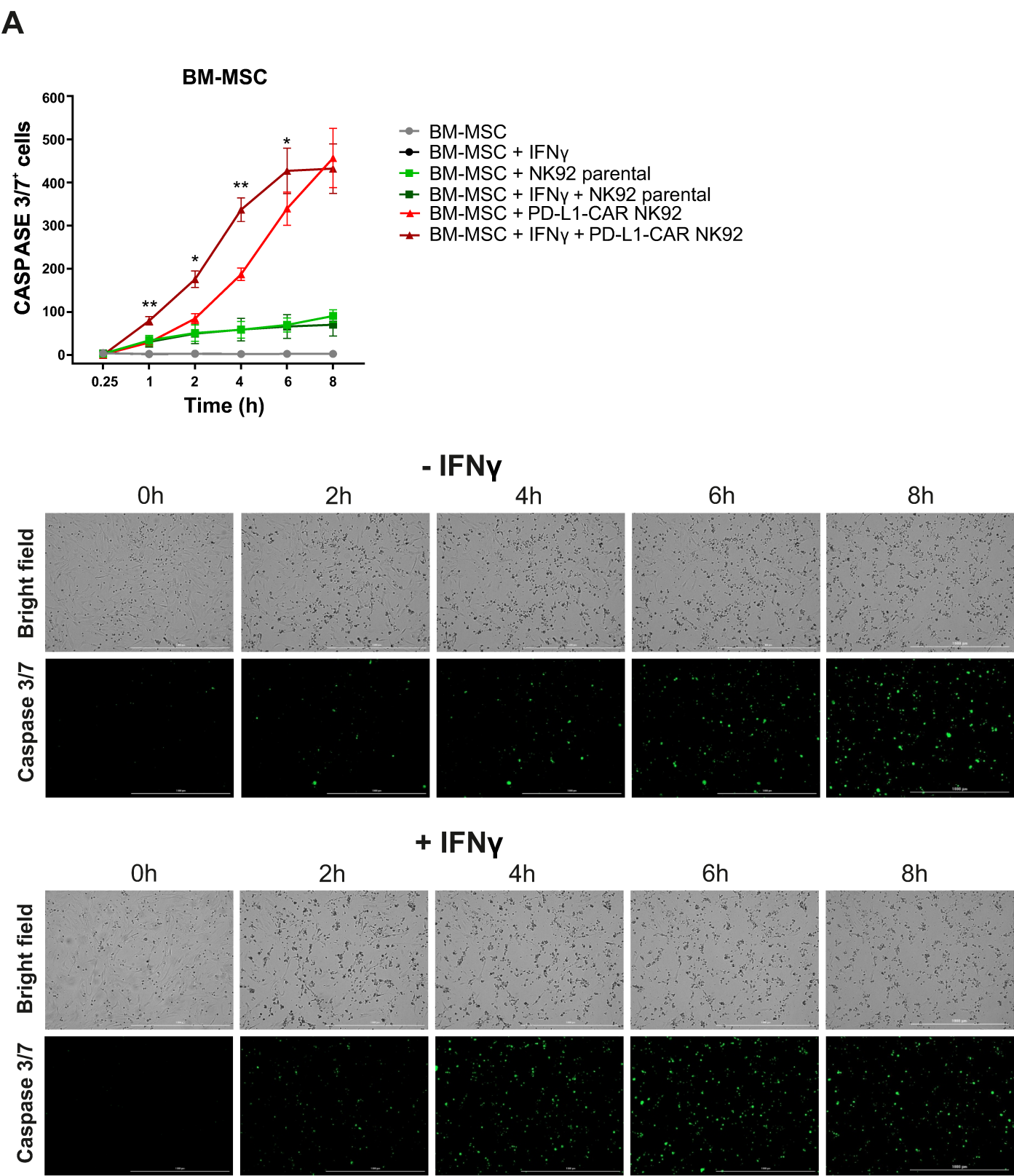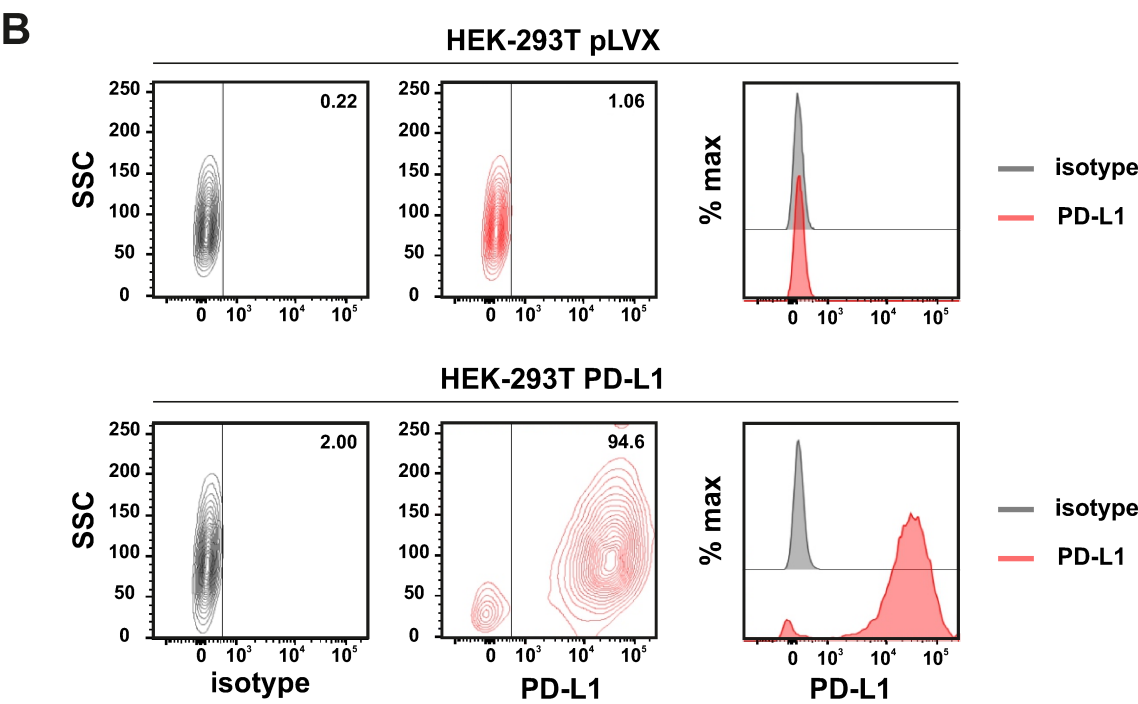

Suppl Fig. 7

Supplement: Supplementary data [file jitc-2021-002500supp002.pdf]
